# Systematic review of the dextral *Hemiplecta* Albers, 1850 (Eupulmonata, Ariophantidae) from Thailand with description of a new species and list of all the Indochinese species

**DOI:** 10.3897/zookeys.1047.65735

**Published:** 2021-06-28

**Authors:** Chirasak Sutcharit, Somsak Panha

**Affiliations:** 1 Animal Systematics Research Unit, Department of Biology, Faculty of Science, Chulalongkorn University, Bangkok 10330, Thailand Chulalongkorn University Bangkok Thailand; 2 Academy of Science, The Royal Society of Thailand, Bangkok 10300, Thailand Academy of Science, The Royal Society of Thailand Bangkok Thailand

**Keywords:** Conservation, edible snails, lectotype, Southeast Asia, taxonomy type specimen

## Abstract

The genus *Hemiplecta* is a group of large-sized land snails which have long been used as a food resource by Indochinese people. There are five dextral and four sinistral species currently recognized from Thailand. The dextral group is comprised of two previously recorded species (*H. humphreysiana* and *H. distincta*), two newly recorded species (*H. funerea* and *H. esculenta*), and one new species (*H. nemorosa***sp. nov.**) from northern Thailand is being proposed. We reassessed the diagnostic characters of the genitalia, mantle edge, and radula. Specimens were classified into the genus *Hemiplecta* on the basis of the penial verge and shell lobe, and on the characters of a bulbous gametolytic sac without a gametolytic duct. A complete species list, together with photographs of the name-bearing types or authenticated specimens and the taxonomic status of *Hemiplecta* s.l. that are known from Indochina including Peninsular Malaysia and Myanmar, is provided for the first time. In total, this species list contains 39 available nominal species names described from this area. Type or authentic specimens can be located for 37 nominal species names, of which 25 are illustrated herein and the other 12 were recently illustrated. However, two available species-level names could not be traced to any type specimens. In addition, lectotypes of *H. funerea* and *H. pluto* are designated herein to stabilize the names.

## Introduction

As currently understood, the diverse ariophantid snail genus *Hemiplecta* Albers, 1850 consists of around 50 dextral species as well as five sinistral species (MolluscaBase 2020; Sutcharit et al. 2021). They are distributed from Indochina through the Sunda Islands to New Guinea (Zilch 1959; Schileyko 2002), with vague records from the Maldives and Kerala, India (Schileyko 2002; Ramakrishna et al. 2010). The genus contains large ariophantid species (shell width up to 70 mm), and at least two species, *H. distincta* (Pfeiffer, 1850) and *H. esculenta* Maassen, 2006, have been known as food for local people in northeastern Thailand, Laos, Cambodia, and Vietnam. It is also an intermediate host of the rat lungworm (Panha 1987b, 1988b, 1994; Maassen 2006; Tesana et al. 2009; Mienis et al. 2010).

Taxonomically, *Hemiplecta* was first established as a distinct section [? subgenus] of *Nanina* Gray, 1834 with a brief definition to contain large helicoid species mainly from the Philippines and the Malay Archipelago (Albers 1850). It was then reassessed with an extended generic description and the addition of several species from India and Southeast Asia (Albers 1860; Godwin-Austen 1888b). Later, it was formally treated as a subgenus of *Nanina* (Adams and Adams 1858; Nevill 1878), but this was not widely accepted. The generic demarcation of *Hemiplecta* was systematically revised based on anatomical characters of its type species by Godwin-Austen (1897). In addition to their shell morphology, species attributed to this genus have a well-developed dart apparatus and a bulbous gametolytic sac without a duct (Godwin-Austen 1897). These characters were accepted as being more reliable than the shell morphology, and were followed until recently (Blanford and Godwin-Austen 1908; Thiele 1931; Zilch 1959; Schileyko 2002). The recent phylogeny of some Indochinese *Hemiplecta* (including the type species) confirm that they are monophyletic and are comprised not only of dextral species but also several sinistral species that were previously included in the *Dyakia* Godwin-Austen, 1891 (see Sutcharit et al. 2021). In addition, the modern systematic revision of various helicarionoid groups has illustrated the taxonomic importance of reproductive characters for distinguishing taxa at both the generic and specific levels (i.e. Hyman and Ponder 2010; Hyman and Köhler 2018, 2019a, b). Until now, the taxonomic treatment of many *Hemiplecta* species has been confusing and remained contentious due to the paucity of crucial reproductive characters.

In Thailand, thirteen nominal (dextral and sinistral) species have previously been attributed to *Hemiplecta*, many of which were uncritically listed in compilations derived from earlier literature (see Panha 1996; Hemmen and Hemmen 2001) and all are based only on shell morphology. Among these, four sinistral species previously attributed to *Dyakia* have been revised and transferred to *Hemiplecta* (see Sutcharit et al. 2021). The other nine dextral species have been re-classified based on analyses of molecular phylogeny and genitalia morphology. Three nominal species, *Helix crossei* Pfeiffer, 1862, *Helix danae* Pfeiffer, 1863a and *Helix weinkauffiana* Crosse & Fischer, 1863, were synonymized and relocated either to *Quantula* Baker, 1941 or *Phuphania*Tumpeesuwan et al., 2007 (Godwin-Austen 1891; Laidlaw 1931, 1933; Hausdorf 1995; Schileyko 2002; Jirapatrasilp et al. 2020). Two other nominal species, *Helix siamensis* Pfeiffer, 1856a and *Hemiplecta dichromatica* Morlet, 1889, were found to possess genitalia with a long gametolytic duct (Maneevong 2000; Boonmachai and Nantarat 2020; Sutcharit et al. 2020; Pholyotha et al. 2021), suggesting that they should be reclassified into the genus *Cryptozona* Mörch, 1872. Therefore, for Thai species, only four dextral species, *H. humphreysiana* (Lea, 1840), *H. distincta*, *H. neptunus* (Pfeiffer, 1861) and *H. zimmayensis* Godwin-Austen, 1888, and four sinistral species are retained in this genus.

In the present study, we aimed to establish a stable and objective taxonomy by incorporating data from the reproductive organs, pallial system and radula morphology. All recognized and undescribed dextral *Hemiplecta* species occurring in Thailand were critically examined, and their morphological variation and distribution ranges are presented. Previously, most of the *Hemiplecta* species have been described based solely on their shells. However, where anatomical data for additional *Hemiplecta* species was available in the literature, this was summarized and compared with the results of the present study. Furthermore, all the nominal taxa currently attributed to the genus *Hemiplecta* s.l. that have the type locality in Indochina, Peninsular Malaysia and Myanmar are alphabetically listed. In addition, the primary type specimens or authentic specimens (when possible) are figured for further comparisons and precise identification.

## Materials and methods

Snails were sampled throughout Thailand. Living snails were euthanized by the two-step method (AVMA 2020), then transferred to 70% (v/v) ethanol for fixation, preservation, and subsequent anatomical study. Genital systems of up to five specimens of each species were examined. Radulae were extracted, and examined under scanning electron microscopy (SEM; JEOL, JSM-5410 LV). Radula shape and teeth formula were analyzed.

**Anatomical abbreviations.** Descriptive terms are oriented with reference to the genital orifice. Abbreviations follow Godwin-Austen (1900, 1919), Schileyko (2002), Pholyotha et al. (2020) and Sutcharit et al. (2021): **ag**, albumin gland; **aldl**, anterior left dorsal lobe; **an**, anus; **at**, atrium; **da**, dart apparatus; **dp**, dart papilla; **e**, epiphallus; **ec**, epiphallic caecum; **fl**, flagellum; **fo**, free oviduct; **gs**, gametolytic sac; **h**; heart; **hd**, hermaphroditic duct; **hg**, hermaphroditic gland; **k**, kidney; **lsl**, left shell lobe; **ov**, oviduct; **p**, penis; **pg**, prostate gland; **pldl**; posterior left dorsal lobe; **pr**, penial retractor muscle; **ps**, penial sculpture; **psh**, penial sheath; **puv**, pulmonary vein; **pv**, penial verge; **r**, rectum; **rdl**, right dorsal lobe; **rsl**, right shell lobe; **ur**, ureter; **v**, vagina; **vd**, vas deferens.

### Institutional abbreviations

**CUMZ** Chulalongkorn University, Museum of Zoology, Bangkok

**FMNH** Field Museum of Natural History, Chicago

**MNHN** Muséum National ďHistoire Naturelle, Paris

**NHM** Natural History Museum, London

**NHMUK** when citing specimens deposited in the **NHM**

**NMNH** National Museum of Natural History, Smithsonian Institute, Washington D.C.

**RMBR** Raffles Museum of Biodiversity Research, Singapore

**RMNH** Naturalis Biodiversity Center, Rijksmuseum van Natuurlijke Historie, Leiden

**SMF** Forschungsinstitut und Naturmuseum Senckenberg, Frankfurt am Main

**UMZC** University Museum of Zoology Cambridge, Cambridge

### Photo credits

Photos of type specimens from the Molluscs Collection (IM) of MNHN are credited to the museum taken under the project E-RECOLNAT: ANR-11-INBS-0004 or as stated otherwise.

## Systematic account

### Family Ariophantidae Godwin-Austen, 1888a

#### Genus Hemiplecta

Taxon classificationAnimaliaStylommatophoraAriophantidae

Albers, 1850

7B583274-9EFD-5408-BE90-5A11F79615A0

Hemiplecta Albers, 1850: 60, 61. Albers 1860: 52, 53. Godwin-Austen 1888b: 155–157. Godwin-Austen 1898: 70, 71. Zilch 1959: 317. Schileyko 2002: 1282, 1283.
Nanina (Hemiplecta) – Adams and Adams 1858: 222. Nevill 1878: 46–48.
Koratia Godwin-Austen, 1919: 202. Type species: Helix distincta Pfeiffer, 1850, by monotypy. Schileyko 2002: 1281, 1282.
Hemiplecta (Koratia) – Zilch 1959: 317. Vaught 1989: 97.
Ariophanta (Semperia) Godwin-Austen, 1898: 82 [non Crosse 1867: 74, 75]. Type species. Helix retrorsa Gould, 1843; by original designation.

##### Type species.

*Helix humphreysiana* Lea, 1840; subsequent designation by Martens in Albers (1860).

##### Diagnosis.

Shell dextral or sinistral, medium to large in size (width about 25 to 75 mm) and monochrome to with stripes, or banding patterns. Apertural lip simple to slightly thickened in adult snails; umbilicus open. Genitalia include penial sheath, straight or coiled epiphallic caecum and short flagellum; penial verge may be present or absent. Dart apparatus well developed; gametolytic sac bulbous to elliptical-shaped (without distinct duct). Mantle edge well developed with or without shell lobes. Jaw smooth (without vertical ribs) and crescentic. Radula with unicuspid central teeth, and bicuspid lateral and marginal teeth.

##### Remarks.

Due to the high degree of similarity in shell morphology, the specific and generic classification within Ariophantidae is usually problematic. There are at least three nominal genera that are often confused, *Nanina* Gray, 1834, *Ariophanta* Des Moulins, 1829 and *Cryptozona* Mörch, 1872. The genitalia have proved to be the distinguishing characters for specific or generic recognition among the Ariophantidae (Laidlaw 1932a; Solem 1966; Sutcharit and Panha 2008). However, only a few species of each genus have been anatomically examined. Based on this limited anatomical information, the unique characters taken from the type species are summarized in Table 1. The genus *Hemiplecta* can be differentiated from these three genera by lacking a gametolytic duct, while the others possess a short to long gametolytic duct. *Hemiplecta* also differs from *Ariophanta* and *Cryptozona* by having unicuspid central teeth, very short or absent flagellum, and a mantle edge with shell lobes; while the latter two genera have tricuspid central teeth, long flagellum, and a mantle edge without shell lobes. In addition, *Hemiplecta* can be distinguished from *Nanina* by its short flagellum (Table 1).

**Table 1.** Comparison of shell and genitalia characters among four genera of Ariophantidae, mainly based on the characteristics of the type species. The superscript numbers are the references: 1 = Schileyko (2002), Thiele (1931), Zilch (1959), 2 = Blanford and Godwin-Austen (1908), 3 = Wiegmann (1898), Sarasin and Sarasin (1899), 4 = Godwin-Austen (1899), and 5 = Sutcharit et al. (2021), present study.

**Characters**	***Ariophanta* Des Moulins, 1829^1,2^**	***Nanina* Gray, 1834^1,3^**	***Hemiplecta* Albers, 1850^1,2,5^**	***Cryptozona* Mörch, 1872^1,4^**
Gametolytic duct	Short	long	absent	short / absent
Gametolytic sac	Bulbous	bulbous	bulbous	bulbous
Epiphallus	short, nearly absent	long	long	long
Epiphallic caecum	long and straight	short and straight	long and straight or coiled	long and straight
Flagellum	very short or absent	long	short	short
Shell lobe	Absent	present	present / absent	absent
Shell coiling	dextral / sinistral	dextral	dextral / sinistral	dextral
Central teeth	Tricuspid	unicuspid	unicuspid / tricuspid	tricuspid
Distribution	South Asia	Indonesia, New Guinea	Southeast Asia, New Guinea	South Asia, Indochina
Type species	*Helix laevipes* Müller, 1774	*Helix citrina* Linnaeus, 1758	*Helix humphreysiana* Lea, 1840	*Helix ligulata* Férussac, 1819

###### Dextral species in Thailand

#### Hemiplecta humphreysiana

Taxon classificationAnimaliaStylommatophoraAriophantidae

(Lea, 1840)

BCA141BA-0932-57C2-9651-C7B503DF8A09

**Figure 1.**
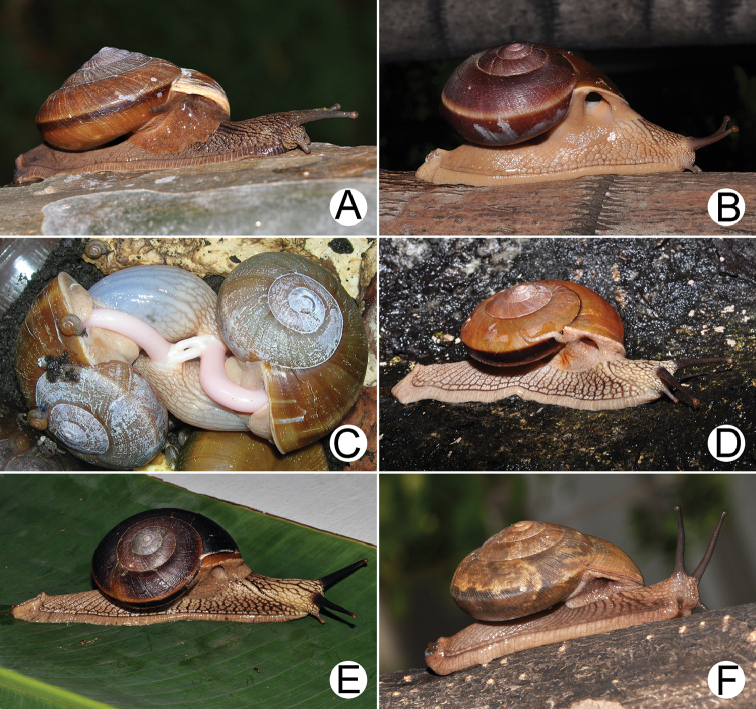
Living snail. **A***Hemiplecta humphreysiana* from Singapore (width about 45 mm) **B, C***Hemiplecta distincta***B** from Saraburi, Thailand (width about 65 mm) and **C** mating pairs **D, E***Hemiplecta funerea* from Nan, Thailand (width about 50 mm) **D** yellow shell form and **E** dark shell form (width about 50 mm) **F***Hemiplecta esculenta* from Chiang Mai, Thailand (width about 30 mm).

**Figure 2.**
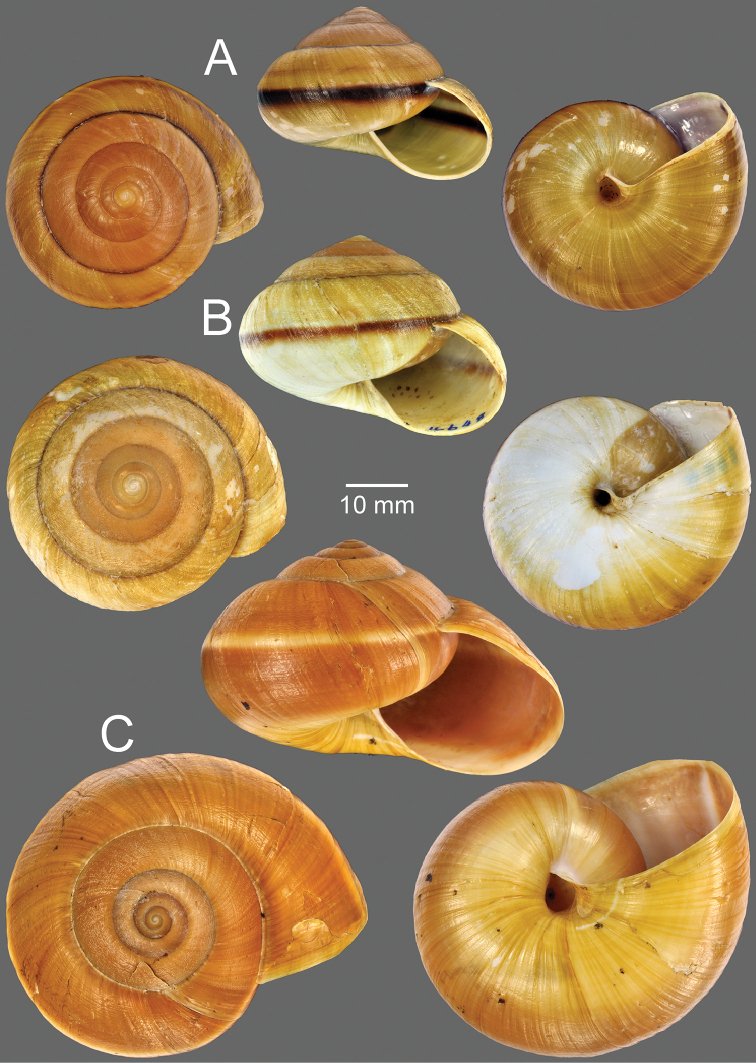
**A, B***Hemiplecta humphreysiana***A** specimen CUMZ 4573 from Singapore and **B** specimen CUMZ 4648 from Narathivat, Thailand **C***Hemiplecta distincta*, specimen CUMZ 4531 from Chacheongsao, Thailand.

**Figure 3.**
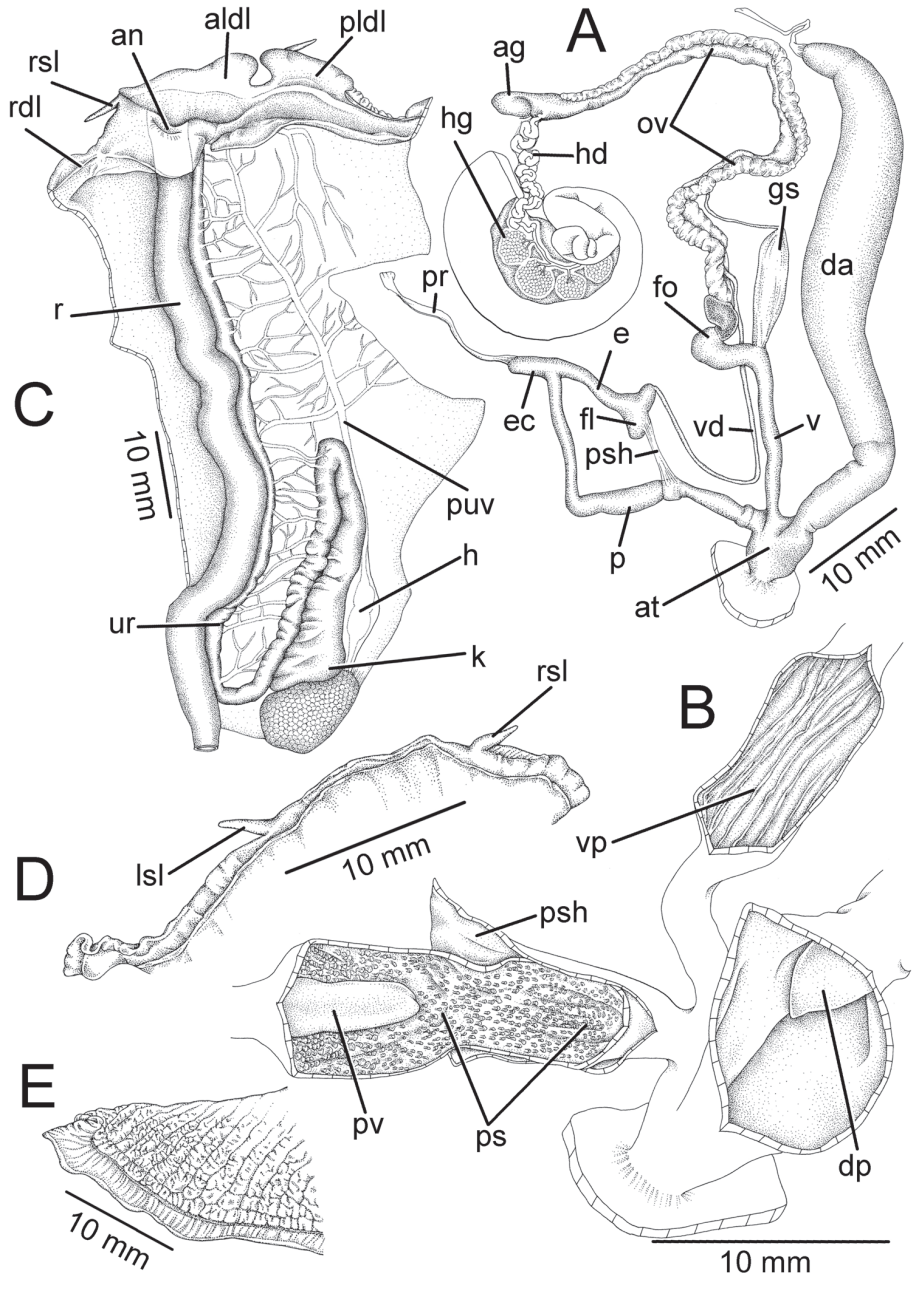
Genitalia, pallial system, mantle edge structure, and caudal region of *Hemiplecta humphreysiana*, specimen CUMZ 4573 from Singapore **A** whole genital organs **B** internal wall sculpture of penis, vagina and dart chamber **C** pallial system, lung cavity and ventral view of mantle edge **D** dorsal view of mantle edge showing shell lobes and **E** right view of caudal region.

Figures 1A , 2A, B , 3 , 10A–C

Helix humphreysiana Lea, 1840: 175. Type locality: Pondicherry and Singapore. Lea 1841: 463, 464, pl. 12, fig. 16. Reeve 1854: Helix pl. 74, species 387.
Hemiplecta humphreysiana : Morgan, 1885a: 378. Godwin-Austen 1898: 74, pl. 80. figs 6, 6b; pl. 61, figs 1, 1e. Collinge 1902: 78, pl. 4, figs 16–23. Laidlaw 1932a: 78. Laidlaw 1932b: 40. Laidlaw 1933: 217. Benthem Jutting 1949: 69. Benthem Jutting 1950: 444, fig. 64. Laidlaw 1957: 134. Benthem Jutting 1959: 148–150. Ho 1995: 104, 105.
Nanina humphreysiana : Martens, 1867: 233, pl. 10, figs 2, 2b, 4. Tryon, 1886: 36, pl. 11, figs 52, 53, pl. 12, fig. 54.
Nanina (Hemiplecta) humphreysiana : Tryon, 1886: 36, pl. 11, figs 52, 53; pl. 12, fig. 54.

##### Type specimen.

See the species list of the Indochinese species (Fig. 12D).

##### Material examined.

**Singapore**: Bukit Timah: RMBR 1990.1711 (1 specimen in ethanol), 1990.15781–2 (2 specimens in ethanol); CUMZ 4573 (1 shell; Figs 2A, 3). Botanic Garden: RMBR 1975.2.10.89 (1 shell), RMBR 1990.1710 (1 specimen in ethanol); CUMZ 4571/1 (1 shell), CUMZ 4572 (4 specimens in ethanol). Nee Soon: RMBR 1990.15945 (1 shell), RMBR 1990.16996 (1 specimen in ethanol), RMBR 1990.15103–4 (2 specimens in ethanol), RMBR 1992.3159 (1 specimen in ethanol), RMBR 1992.3160–1 (2 shells), RMBR 1992.3162 (1 specimen in ethanol), RMBR 1994.4116 (1 specimen in ethanol). Singapore: RMBR 1989.509–513 (5 shells), RMBR 1990.15105 (1 specimen in ethanol). **Thailand**: Sirindhorn Waterfall, Halabala National Park, Narathivat Province: CUMZ 4647 (2 shells), CUMZ 4648 (1 shell; Fig. 2B).

***Shell.*** Shell large (height up to 40 mm, width up to 55 mm), dextral and conic to depressed conic (Fig. 2A, B). Whorls 6 to 8, slightly convex; suture wide and shallow. Shell yellowish to brownish, usually with narrow dark brown band on periphery. Upper shell surface darker than lower surface. Apex obtuse; embryonic shell large and smooth; following whorls with thin growth lines. Last whorl rounded to slightly angulate; aperture ovate; lip simple but slightly thickened in adult snail. Columella slightly dilated; parietal callus thin and translucent. Umbilicus open and deep.

***Genitalia.*** Atrium (at) very short (Fig. 3A). Penis (p) long, slender, cylindrical, and encircled by thin penial sheath (psh) extending about one-third of penis length. Epiphallic caecum (ec) short, straight; penial retractor muscle (pr) thin and attached to the tip. Epiphallus (e) short and about one-third of penis length. Flagellum (fl) short, stout, and with thin muscle bands connected to penial sheath. Vas deferens (vd) small tube. Internal wall of penis with sculpture encircling penial verge (Fig. 3B). Penial sculpture (ps) consists of scattering of small papillary knobs arranged randomly on penial wall. Penial verge (pv) long conic with smooth surface.

Vagina (v) long and cylindrical (Fig. 3A, B); internal wall with thin and smooth longitudinal vaginal pilasters (vp). Dart apparatus (da) long muscular cylinder, externally and internally smooth; dart papilla (dp) short, conic, and smooth. Gametolytic sac (gs) elongate or bulbous without distinct duct. Free oviduct (fo) long and encircled with thickened blackish muscular tissue (orange in fresh specimens). Oviduct (ov) long and with lobules; prostate gland bound to oviduct. Albumen gland (ag) small. Hermaphroditic duct (hd) small, convoluted, and connected to lobules of hermaphroditic gland (hg).

***Radula.*** Each row containing about 253 teeth (127–(18–32)–1–(29–32)–125). Central tooth unicuspid and triangular (Fig. 10A). Lateral teeth unicuspid, slanted, and with curved cusp. Outer lateral teeth with slightly curved cusps; latero-marginal transition from tooth numbers 28 to 32 (Fig. 10B). Marginal teeth bicuspid and curved; endocone and ectocone usually similar in shape and size (Fig. 10C).

***External features*.** Mantle edge with large dorsal lobes. Right dorsal lobe (rdl) to right of anus (an; on the left in figure), large, and thick. Left dorsal lobe to left of anus (on the right in figure), composed of thin crescentic anterior left dorsal lobe (aldl), and thin elongated posterior left dorsal lobe (pldl). Right shell lobe (rsl) and left shell lobe (lsl) have short finger-shaped extensions located on mantle edge near tip of urinary groove and around junction of anterior and posterior left dorsal lobes, respectively, (Fig. 3C, D).

Pulmonary cavity typically sigmurethran, heart (h; auricle and ventricle) located left of kidney (k; on the right in figure). Pulmonary cavity approximately four times longer than wide. Pulmonary vein (puv) and venation on lung cavity well developed and distinct. Kidney (k) elongate, slender, and approximately one-third length of pulmonary cavity. Ureter (ur) sigmoid, closed tube arising from tip of kidney, extending along right side of kidney, and curved adjacent to rectum (r). Anus (an) adjacent to mantle edge (Fig. 3C).

Living snails possess long greyish-brown tentacles (Fig. 1A). Skin reticulated brownish with blackish reticulations around head. Foot sole relatively elongate, broad and unipartite. Sole of foot plain brownish; side of body brownish; upper part of tail dark greyish. Tail long, curved mid-dorsally, tall dome-shaped in cross section. Caudal horn not overhanging; caudal foss a long vertical slit arranged on tail above sole margin. Pedal groove typical aulacopod and well defined (Fig. 3E).

##### Distribution.

The systematic studies of some *Hemiplecta* species have revealed incongruence between the traditional shell-based and molecular classifications (Sutcharit et al. 2021). Therefore, apart from Singapore (type locality) the historical record of *H. humphreysiana* from Sumatra, Borneo, and several localities in Peninsular Malaysia (Martens 1867; Tryon 1886; Godwin-Austen 1898; Collinge 1902; Laidlaw 1932a, 1933, 1957; Benthem Jutting 1950, 1959) needs to be confirmed by more convincing morphological and molecular evidences. In Thailand, this is the first and only record of this species from Narathivat, the southern-most province of Thailand.

##### Remarks.

The specimens examined and described herein for the genitalia, pallial system, and radula were collected from Singapore (the correct type locality of this type species) to specify the characteristics for the genus. *Hemiplecta humphreysiana* clearly differs from all other species recorded both in Thailand and Peninsular Malaysia (compared with the type specimens in the list of the species). It can be distinguished from *H. floweri* Smith, 1899 (see Godwin-Austen 1900) from Peninsular Malaysia, by having a narrow umbilicus, without a brownish spiral band on the umbilical area, and more elevated spire (Smith 1898; Godwin-Austen 1900). In addition, the straight epiphallic caecum of this species is distinct from the coiled epiphallic caecum of *H. floweri* (Table 2). Unfortunately, none of the penial sculptures have been prepared for further comparison.

**Table 2.** Shell coiling, shell lobe, and genitalia variation among species that have been classified into the genus *Hemiplecta* s.l.: +, present; -, absent; ? not shown in the literature. References: 1 = Benthem Jutting (1950), 2 = Blanford and Godwin-Austen (1908), 3 = Collinge (1902), 4 = Godwin-Austen (1898), 5 = Godwin-Austen (1900), 6 = Godwin-Austen (1919), 7 = Maassen (2009), 8 = Panha (1987a), 9 = Schileyko (2002), 10 = Schileyko (2015), 11 = Stoliczka (1873), 12 = Sutcharit et al. (2012), and 13 = Sutcharit et al. (2021).

Taxa	Shell coiling	Right/left shell lobes	Epiphallic caecum	Penial verge	Dart apparatus	Gametolytic duct	References
*H. abbasi* Maassen, 2009	dextral	?	straight	+	+	-	7
*H. ceylanica* (Pfeiffer, 1850)	dextral	?	straight	+	+	-	9
*H. cymatium* Pfeiffer, 1856)	dextral	+/+	straight	+	+	-	11
*H. distincta* (Pfeiffer, 1850)	dextral	-/-	straight	-	+	-	5, 6, 8, 9 and present study
*H. esculenta* Maassen, 2006	dextral	-/-	straight	+	+	-	present study
*H. floweri* (Smith, 1899)	dextral	+/+	coiled	+	+	-	5
*H. funerea* (Smith, 1896)	dextral	+/-	coiled	-	+	-	present study
*H. humphreysiana* (Lea, 1840)	dextral	+/+	straight	+	+	-	1, 2, 3, 4 and present study
*H. ligorica* Sutcharit & Panha, 2021	sinistral	-/-	straight	-	+	-	13
*H. nemorosa* sp. nov.	dextral	-/-	straight	+	+	-	present study
*H. pernobilis* (Férussac, 1821)	dextral	?	coiled	-	+	-	10
*H. retrorsa* (Gould, 1843)	sinistral	-/-	straight	-	+	-	13
*H. salangana* (Martens, 1883)	sinistral	-/-	straight	-	+	-	12
*H. thailandica* Sutcharit & Panha, 2021	sinistral	-/-	straight	-	+	-	13
**Doubtful generic status**							
*H. malaouyi* (Morgan, 1885)	dextral	+/+	coiled	?	+	long cylindrical	Laidlaw (1932a)
*H. densa* (Adams & Reeve, 1850)	dextral	+/+	straight	?	+	long cylindrical	Wiegmann (1898)
*H. werberi* (Sarasin & Sarasin, 1899)	dextral	?	straight	?	+	long cylindrical	Niethammer (1937)
*H. foersteri* Kobelt, 1914	dextral	?	straight	?	-	long cylindrical	Wiktor (2003)
*H. belerang* Cilia & Abbas, 2012	dextral	?	straight	+	+	long cylindrical	Cilia and Abbas (2012)

#### Hemiplecta distincta

Taxon classificationAnimaliaStylommatophoraAriophantidae

(Pfeiffer, 1850)

E80A2E8D-C02D-5D24-8AF3-07DD2235C9DF

**Figure 4.**
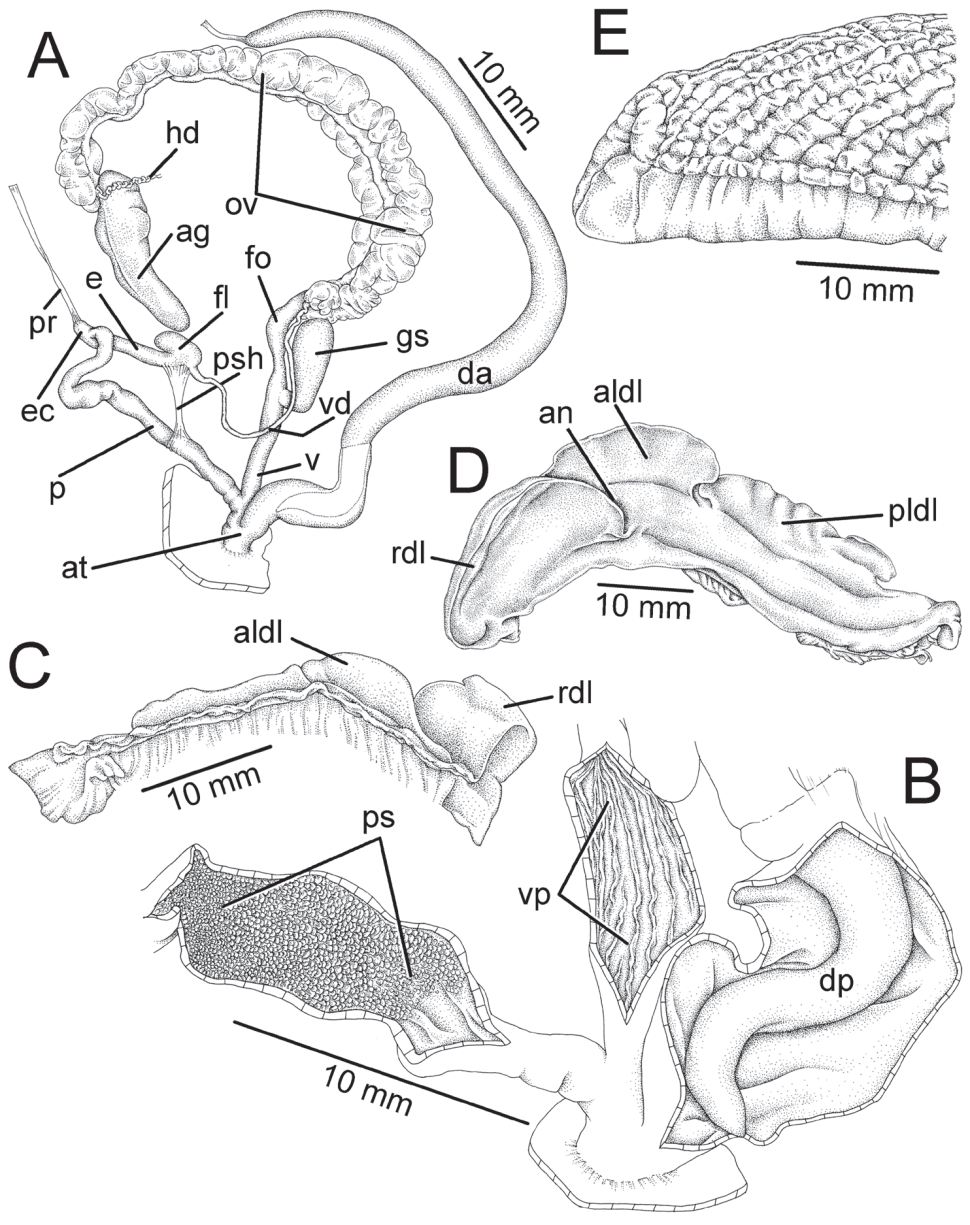
Genitalia, mantle edge structure, and caudal region of *Hemiplecta distincta*, specimen CUMZ 4560 from Chanthaburi, Thailand **A** whole genital organ **B** internal wall sculpture of penis, vagina, and dart chamber **C** dorsal view of mantle edge **D** ventral view of mantle edge and **E** right view of caudal region.

Figures 1B, C , 2C , 4 , 10D, E

Helix distincta Pfeiffer, 1850: 69, 70. Type locality: Insulis Moluccis [possibly error or mislabeling]. Pfeiffer 1853: 346, pl. 134, figs 1, 2. Reeve 1854: Helix pl. 86, species 465.
Nanina distincta : Martens, 1860: 7.
Helix neptunus Pfeiffer, 1861a: 190. Type locality: Siam [Thailand]. Pfeiffer 1861b: 176, 177. pl. 48, figs 1, 2. New synonym
Nanina (Rhyssota) distincta : Martens, 1867: 69, 70, pl. 6, fig. 8.
Nanina (Hemiplecta) distincta : Tryon, 1886: 30, pl. 8, fig. 26.
Nanina (Hemiplecta) neptunus : Tryon, 1886: 34, pl. 8, fig. 27.
Hemiplecta zimmayensis Godwin-Austen, 1888c: 241, 242. Type locality: Zimme, Siam territory [Chiang Mai Province, Thailand]. New synonym
Ariophanta (Hemiplecta) distincta : Morelet, 1891: 231.
Hemiplecta distincta : Morelet, 1889: 124. Blanford 1903: 277, 278. Panha 1987a: 108–115, figs 1–3. Panha 1987b: 25–34, fig. 9. Panha 1988a: 197–206, figs 6, 7. Panha 1988b 233–239. Panha 1994: 4–15. Inkhavilay et al. 2019: 76, figs 35b, c, 56c.
Nanina (Rhysota) distincta : Fischer and Dautzenberg 1904: 393. Dautzenberg and Fischer 1906: 346, 347.
Nanina (Rhysota) distincta var. neptunus : Dautzenberg and Fischer 1908: 171.
Koratia distincta : Godwin-Austen 1919: 199–202, figs 1, 2. Schileyko 2002: 1282, 1283, fig. 1685.
Nanina (Rhysota) distincta neptunus : Dautzenberg and Fischer, 1906: 347, 348.
Hemiplecta (Koratia) distincta : Solem, 1966: 27.
Hemiplecta (Hemiplecta) distincta : Hemmen and Hemmen, 2001: 44, fig. 12.
Hemiplecta (Hemiplecta) neptunus : Hemmen and Hemmen, 2001: 44.
Hemiplecta (Hemiplecta) zimmayensis : Hemmen and Hemmen, 2001: 44.

##### Type specimen.

See the species list of Indochinese species (Fig. 11C).

##### Material examined.

**Thailand**: Tam Chiang Dao, Chiang Mai: CUMZ 4550 (1 shell), CUMZ 4558 (5 shells). Wang Chao Waterfall, Kampangphet: CUMZ 4641 (4 shells). Klong Lann National Park, Kampangphet: CUMZ 4579 (2 specimens in ethanol). Kaeng Jed Kwae, Watbot, Phitsanuloke: CUMZ 4638 (7 shells). Tam Wang Daeng, Nern Maprang, Phitsanuloke: CUMZ 4632 (1 shell). Khao Nang Rum, Huay Kla Klang National Park, Uthaithani: CUMZ 4502 (6 shells), CUMZ 4510 (3 shells), CUMZ 4538 (3 shells), CUMZ 4541 (6 shells), CUMZ 4607 (6 shells), CUMZ 4610 (9 shells), CUMZ 4611 (3 shells). Jed Sow Noi Waterfall, Muaklek, Saraburi: CUMZ 4548 (3 shells). Pu Kare Botanic Garden, Saraburi: CUMZ 4505 (4 shells), CUMZ 4506 (1 shell), CUMZ 4534 (5 shells). Tam Dao Khao Kaew, Muaklek, Saraburi: CUMZ 4624 (1 shell). Wat Tharahat, Saraburi: CUMZ 4508 (1 shell), 4530 (1 shell). Sam Larn National Park, Saraburi: CUMZ 4578 (2 specimens in ethanol). Bang Srithong, Bang Kruay, Nonthaburi: CUMZ 4555 (5 shells). Khao Look Chang, Pakchong, Nakhonratchasima: CUMZ 4501 (8 shells), CUMZ 4606 (9 shells), CUMZ 4612 (9 shells), CUMZ 4535 (3 shells). Tub Lann National Park, Nakhonratchasima: CUMZ 4617 (1 shell). Nawang, Nongbualumphu: CUMZ 4529 (1 shell). Tam Suwankuha, Nongbualumphu: CUMZ 4633 (3 shells), 4637 (3 shells). Thung Kra-Mang, Phu Kiew Wildlife Sanctuary, Chaiyaphum: CUMZ 4608 (5 shells). Pang Khone, Sakonnakhon: CUMZ 4619 (4 shells). Phuphan Mountains, Sakonnakhon: CUMZ 4504 (6 shells), CUMZ 4507 (5 shells). Phu Kum Khao, Sahatsakhan, Kalasin: CUMZ 4557 (8 shells). Phu Sri Tharn Wildlife Sanctuary, Kalasin: CUMZ 4621 (1 shell). Huay Lao Waterfall, Phuluang Wildlife Sanctuary, Loei: CUMZ 4634 (1 shell). Tam Pha Bend, Chiang Karn, Loei: CUMZ 4532 (1 shell). Tam Pha Bing, Wangsapung, Loei: CUMZ 4636 (1 shell). Tam Piya, Loei: CUMZ 4639 (3 shells). Tam Mahasombat, Lomsak, Phetchabun: CUMZ 4567 (1 shell). Tam Yai Namnao, Namnao National Park, Phetchabun: CUMZ 4566 (1 shell), CUMZ 4622 (1 shell). Tam Phraya Nakarat, Phuphaman National Park, Khonkaen: CUMZ 4635 (1 shell). Pha Tam National Park, Ubonratchathani: CUMZ 4604 (3 shells), CUMZ 4616 (3 shells). Yod Dome National Park, Buriram: CUMZ 4629 (2 shells). Wang Ta Krai Waterfall, Nakhonnayok: CUMZ 4540 (2 shells), CUMZ 4549 (2 shells), CUMZ 4605 (1 shell), CUMZ 4640 (5 shells), CUMZ 4577 (1 specimen in ethanol). Khao Ang Rue Nai Wildlife Sanctuary, Chachoengsao: CUMZ 4531 (1 shell; Fig. 2B), CUMZ 4546 (7 shells), CUMZ 4609 (3 shells), CUMZ 4613 (4 shells), CUMZ 4620 (2 shells), CUMZ 4627 (1 shell), CUMZ 4630 (1 shell). Pang Srida National Park, Prachinburi: CUMZ 4631 (4 shells). Ra-Ru, Taphraya, Srakeow: CUMZ 4628 (1 shell). Tam Leum, Klonghad, Srakeow: CUMZ 4625 (1 shell). Khao Cha Ang-Oan, Borthong, Chonburi: CUMZ 4542 (4 shells), CUMZ 4618 (1 shell), CUMZ 4626 (4 shells). Khao Cha Mao Waterfall, Rayong: CUMZ 4543 (1 shell). Tam Suwanphupha, Khao Chamao, Rayong: CUMZ 4545 (1 shell). Wat Ma-deau (Tam Khao Loi), Khao Chamao, Rayong: CUMZ 4544 (4 shells). Plieu National Park, Chanthaburi: CUMZ 4509 (1 shell), CUMZ 4536 (1 shell), CUMZ 4539 (9 shells), CUMZ 4560 (1 specimen in ethanol; Fig. 4), CUMZ 4601 (1 shell), CUMZ 4615 (4 shells). Sibha Shan Waterfall, Chanthaburi: CUMZ 4547 (6 shells). Tha Mai District, Chanthaburi: CUMZ 4603 (1 shell). Koh Kud, Trat: CUMZ 4559 (9 shells), CUMZ 4614 (7 shells). Kaeng Kracharn National Park, Phetchaburi: CUMZ 4527 (1 shell). Tam Nam Pud, Pangnga: CUMZ 4623 (1 shell).

***Shell.*** Shell large (height up to 55 mm, width up to 75 mm), yellowish with white narrow peripheral band, and paler color below on lower shell surface. Upper shell surface with thin growth lines interrupted with spiral wrinkles. Last whorl large and rounded; aperture large ovate; lip simple but slightly thickened in adult snails (Fig. 2C).

***Genitalia.*** The external genital organs were described in Godwin-Austen (1900, 1919). Gametolytic sac (gs) bulbous with undifferentiated duct. Internal wall of penis exhibits closely packed papilla knobs that abruptly cease near atrium; penial verge absent. Internal sculpture of vagina with thin and smooth longitudinal vaginal pilasters (vp). Internal surface of dart apparatus smooth; dart papilla (dp) conic, and with a smooth surface (Fig. 4A, B).

***Radula.*** Each row with about 543 teeth (276–(15–20)–1–(15–20)–276). Central tooth unicuspid triangular with rounded head (Fig. 10D). Lateral teeth unicuspid, oblique and triangular. Outer lateral teeth unicuspid, sickle-shaped, with transition to curved and narrow sickle form; latero-marginal transition starts from tooth numbers 15 to 20. Marginal teeth narrowly curved, bicuspid; endocone and ectocone small and pointed (Fig. 10E).

***External features.*** Living snails have a similar soft body morphology and pulmonary cavity to that of *H. humphreysiana*. The distinct characters are pale brown to brownish body (Fig. 1B, C). Sole of foot brownish; caudal horn not overhanging; caudal foss a long vertical slit arranged on tail above sole margin. Pedal groove typical aulacopod and well defined (Fig. 4E). Mantle edge narrow with large dorsal lobes. Right dorsal lobe (rdl) to right of pneumostome, large and thick; left dorsal lobe to left of pneumostome, composed of anterior left dorsal lobe (aldl) and posterior left dorsal lobe (pldl); shell lobe absent (Fig. 4C, D).

##### Distribution.

Ranges from Cambodia to Laos, Thailand and southern Vietnam (Smith 1896; Laidlaw 1932a; Panha 1988a, 1994; Schileyko 2011; Inkhavilay et al. 2019). In Thailand, *H. distincta* is fairly abundant and occurs throughout the country, except for southern Thailand (Panha 1988b, 1994). The southern limit of the species appears to be near the Isthmus of Kra (10°N). We have a single and old shell from Pangnga Province, southern Thailand that needs to be confirmed.

##### Remarks.

The type specimens of *Helix neptunus* Pfeiffer, 1861 and *Hemiplecta zimmayensis* Godwin-Austen, 1888 exhibit a shell morphology and color patterns identical to that of *H. distincta*. The absence of a whitish peripheral band in *Helix neptunus* Pfeiffer, 1861 and the strong growth lines of *Hemiplecta zimmayensis* are the only observed differences from *H. distincta*. Therefore, we recognize these two nominal species as junior subjective synonyms of *H. distincta*.

*Hemiplecta distincta* has long been considered a food item for local people in northeastern Thailand (Panha 1987b), as well as in Cambodia and Laos (personal observation). It has also been found to be an intermediate host of the rat lungworm, a human pathogen (Panha 1987b, 1988b). The life cycle and breeding biology of this species have been extensively studied (Panha 1987a, b, 1988a, b, 1994).

#### Hemiplecta funerea

Taxon classificationAnimaliaStylommatophoraAriophantidae

(Smith, 1896)

18ACEFA5-526C-5479-89C7-73C01B4AEDA7

**Figure 5.**
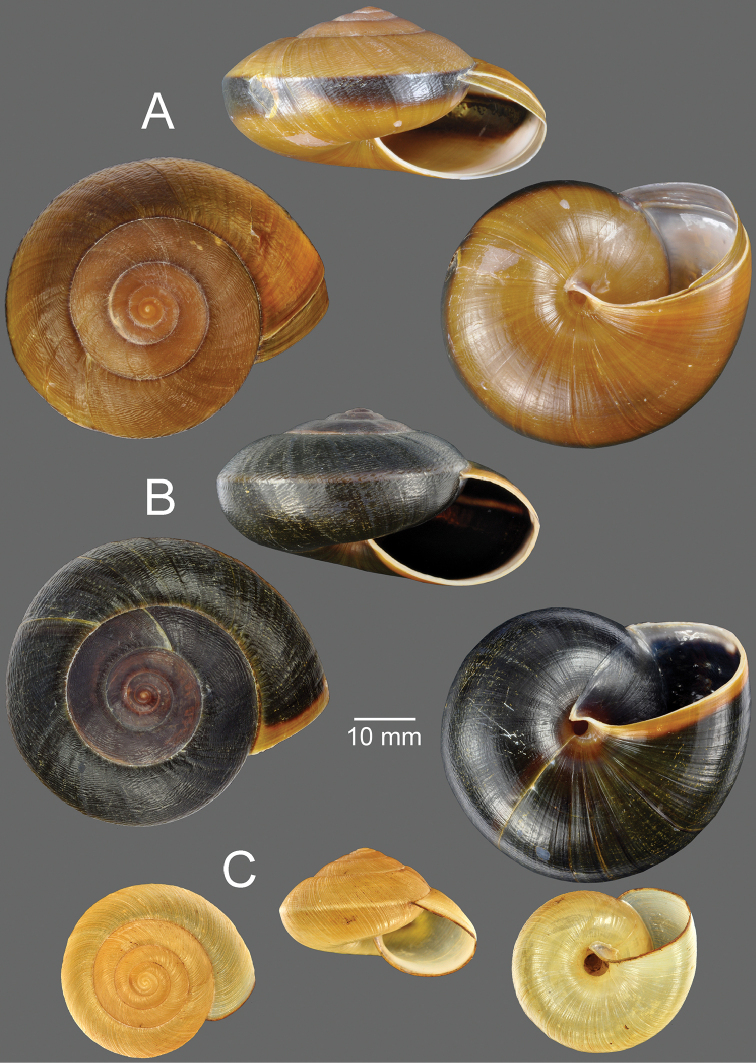
**A, B***Hemiplecta funerea*, specimens CUMZ 4575 from Nan, Thailand **A** yellow shell form and **B** dark shell form **C***Hemiplecta esculenta*, specimen CUMZ 4553 from Chiang Mai, Thailand.

**Figure 6.**
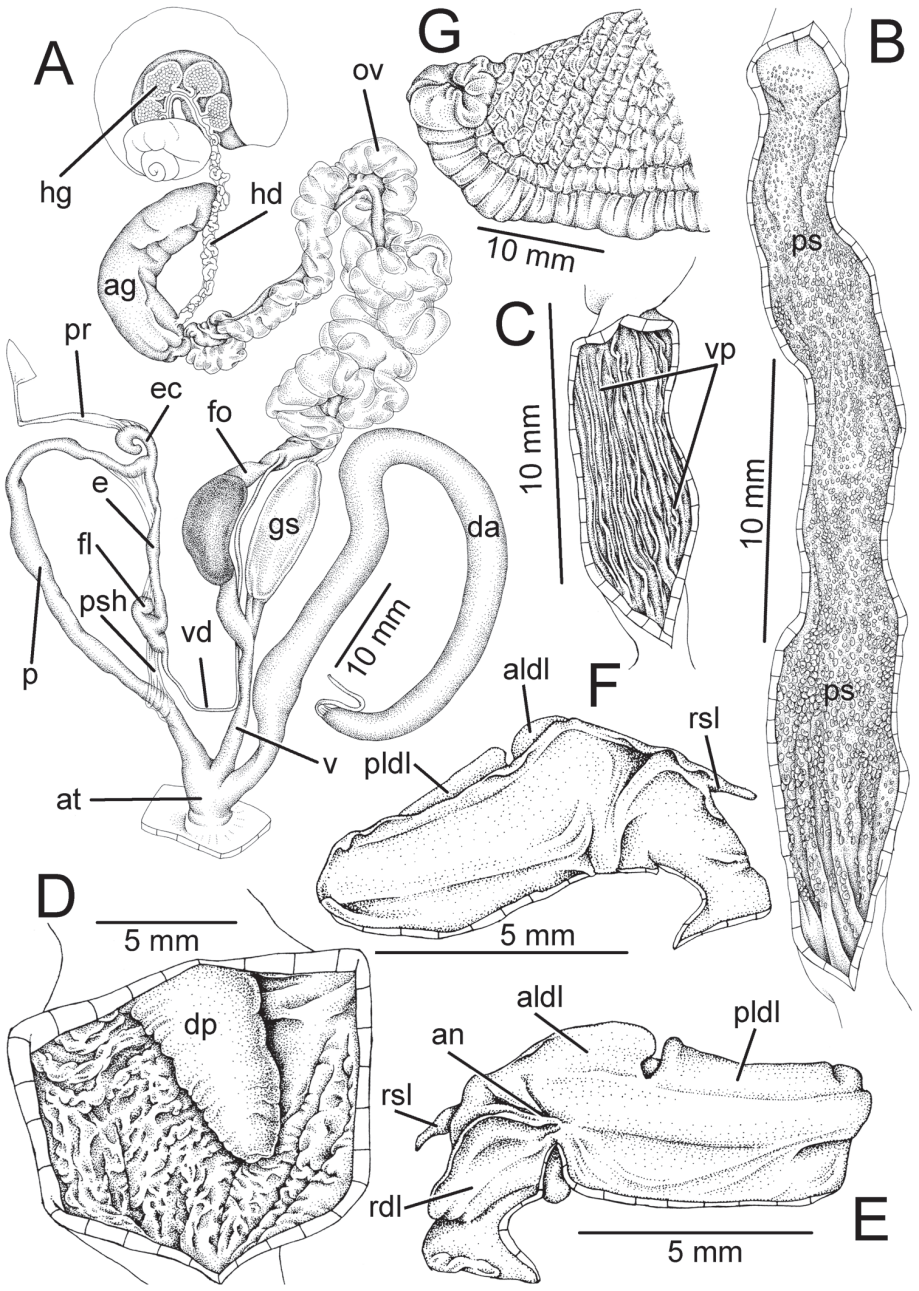
Genitalia, mantle edge structure and caudal region of *Hemiplecta funerea*, specimen CUMZ 4575 from Nan, Thailand **A** whole genital organs **B** internal wall sculpture of penis **C** internal wall sculpture of vagina **D** internal wall sculpture of dart chamber **E** ventral view of mantle edge **F** dorsal view of mantle edge showing right shell lobe and **G** right view of caudal region.

Figures 1D, E , 5A, B , 6 , 10F, G

Nanina distincta var. funerea Smith, 1896: 128. Type Locality: Vanbu, Tonkin [Van Ban District, Lao Cai Province, Vietnam]. Fischer and Dautzenberg 1904: 393.
Nanina distincta var. pallidior Smith, 1896: 128. Type Locality: Vanbu, Tonkin [Van Ban District, Lao Cai Province, Vietnam]. Fischer and Dautzenberg 1904: 393.
Hemiplecta funerea : Inkhavilay et al. 2019: 76, 77, figs 35f, 36a.

##### Type specimen.

See the species list of Indochinese species (Fig. 12B).

##### Material examined.

**Thailand**: Bor Klue District, Nan: CUMZ 4649 (5 shells). Ton Tong Waterfall, Doi Phu Ka National Park, Nan: CUMZ 4575 (8 specimens in ethanol; Figs 1D, E, 5A, B), CUMZ 4576 (8 shells).

***Shell.*** Shell large (height up to 35 mm, width up to 55 mm), depressed conic, dextral, with 6–7 whorls; spire slightly elevated with wide and shallow suture. Shell almost black to dark brown with thin yellowish peripheral band. Apex obtuse; embryonic shell large with smooth surface; subsequent whorls with thin growth lines and thin radial wrinkles. Last whorl keeled; aperture large and ovate; lip simple, yellowish to dark yellow, and slightly thickened in adult snail. Columella slightly dilated; parietal callus thin and transparent. Umbilicus wide and deep (Fig. 5A, B).

***Genitalia*.** Both male and female genital characters similar to that of *H. humphreysiana*. Gametolytic sac (gs) elongate with undifferentiated duct. The unique characters are a coiled epiphallic caecum (ec) and curved flagellum (fl), which are not found in the other species (Fig. 6A). Internally, penial sculpture (ps) consists of scattered papillary knobs lining penial wall; penial verge absent (Fig. 6B). Internal wall of vagina and internal structure of dart apparatus are similar to that in *H. humphreysiana* (Fig. 6C, D).

***Radula.*** Each row contains about 286 teeth (140–(65–75)–1–(65–75)–135). Central tooth unicuspid conic-shaped, and dull cusp (Fig. 10F). Lateral teeth unicuspid, elongate, and slanted with pointed tip. Outer lateral teeth unicuspid, elongate; latero-marginal transition starts from tooth numbers 65 to 75. Marginal teeth slightly curved, bicuspid; endocone and ectocones small and of similar size (Fig. 10G).

***External features.*** Living snails with long, black eye tentacles (Fig. 1D, E). Skin reticulated, pale brownish to brownish with dark reticulation across the entire head and foot above the lateral margin. Foot sole, caudal foss (Fig. 6G), caudal horn, and pedal groove similar to those in *H. humphreysiana*. Mantle edge, dorsal lobe, and shell lobe similar to those in *H. humphreysiana*, but only long and finger-shaped right shell lobe (rsl) present (Fig. 6E, F).

##### Distribution.

Previously recorded from the type locality in northern Vietnam, and several localities in northern and central Laos (Smith 1896; Fischer and Dautzenberg 1904; Inkhavilay et al. 2019). Recently, we recorded this species from two localities in Nan Province, northern Thailand.

##### Remarks.

Smith (1896: 128) introduced two nominal subspecies of *H. distincta* from northern Vietnam, which were distinguished on the basis of shell color. The “var. funerea” has a purplish-black tinted shell (Fig. 5B), while the “var. pallidior” possesses a yellowish or olive-yellow shell (Fig. 5A). Since then, no new specimens of these two color forms have been critically examined or their status verified. We have collected both living snails and empty shells of these two forms in recent surveys in Laos and Thailand. The two forms are anatomically and genetically identical, and occur syntopically, and thus, in our judgement, are examples of different shell colors of the same species. We recognize *H. funerea* as a distinct and valid species, and treat Nanina distincta var. pallidior Smith, 1896 as its junior synonym (ICZN 1999: Art. 24, 74).

*Hemiplecta funerea* can be distinguished from *H. distincta* by the angulated, dark brown or yellowish shell, distinct penial sculpture, and a long and distinctively coiled epiphallic caecum (Fig. 6A) compared to the short and straight epiphallic caecum in *H. distincta* (Fig. 4A). The blackish reticulated skin on a yellowish background and blackish eye tentacles contrast with the greyish body in *H. distincta* (Fig. 1B–E).

#### Hemiplecta esculenta

Taxon classificationAnimaliaStylommatophoraAriophantidae

Maassen, 2006

34F9EB4C-C4FD-593E-9C6E-7E720F72E575

**Figure 7.**
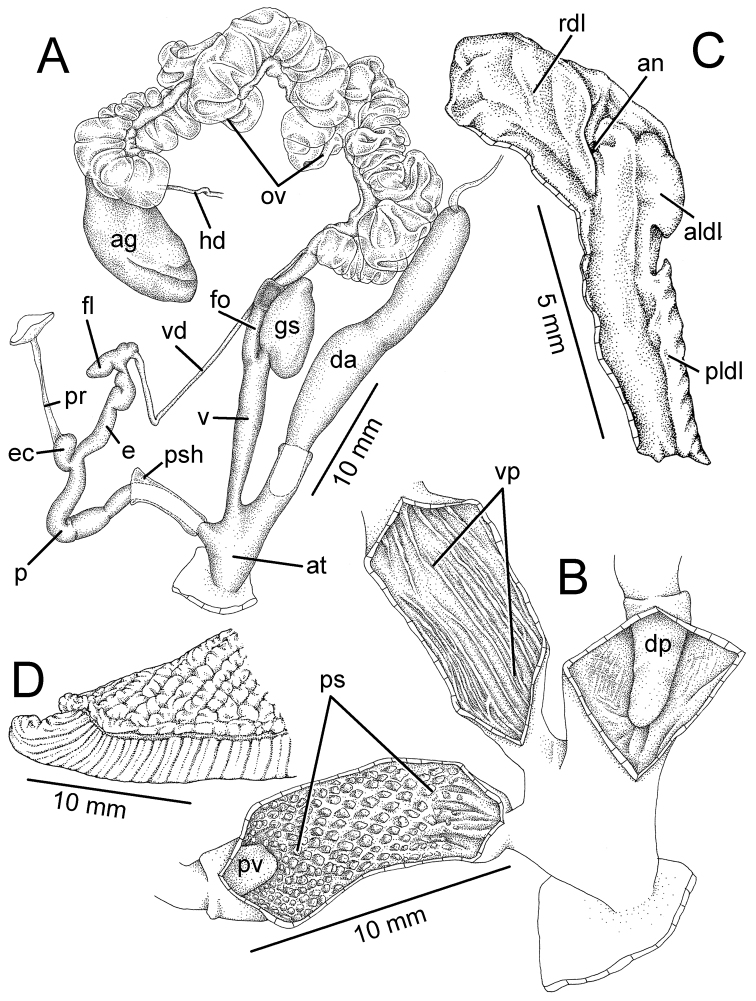
Genitalia, mantle edge structure, and caudal region of *Hemiplecta esculenta*, specimen CUMZ 4553 from Chiang Mai, Thailand **A** whole genital organ **B** internal wall sculpture of penis, vagina and dart chamber **C** ventral view of mantle edge and **D** right view of caudal region.

Figures 1F , 5C , 7 , 10H, I

Hemiplecta esculenta Maassen, 2006: 17, 18, figs 10–12. Type locality: limestone area near village Hang, Pu Luong National Park, Thanh Hoa, Vietnam. Inkhavilay et al. 2019: 76, fig. 35d, e. Páll-Gergely 2019: figs 11–13.

##### Type specimen.

See the species list of Indochinese species.

##### Material examined.

**Thailand**: Tam Chiang Dao, Chiang Mai: CUMZ 4553 (5 specimens in ethanol; Fig. 1F), CUMZ 4564 (10 specimens in ethanol), CUMZ 4565 (9 specimens in ethanol), CUMZ 4574 (8 specimens in ethanol; Fig. 5C); Tam Tab-Tao, Chaiprakarn, Chiang Mai: CUMZ 4580 (10 specimens in ethanol).

***Shell.*** Shell relatively small (height up to 25 mm, width up to 35 mm), elevated to slightly depressed, upper surface with distinct nodules arranged on growth line, and lower shell surface nearly smooth. Last whorl keeled; aperture large and ovate; lip simple to slightly expanded and dark brown. Umbilicus widely opened and deep (Fig. 5C).

***Genitalia.*** Genital tracts similar to those of *H. humphreysiana* (Fig. 7A). Internal wall of penis with sculpture encircling penial verge. Penial sculpture (ps) consists of small to large papillary knobs arranged in oblique lines on penial wall; relatively smaller knobs surrounding penial verge. Penial verge (pv) small, short, conic, and with smooth surface (Fig. 7B). Gametolytic sac (gs) bulbous with undifferentiated duct. Internal wall of vagina with series of thin longitudinal vaginal pilasters (vp). Dart apparatus (da) relatively short; internal wall of chamber with smooth wall, and papilla of dart apparatus (dp) slightly elongate, conic, and with smooth surface (Fig. 7B).

***Radula.*** Each row possesses about 161 teeth (80–(18–21)–1–(19–21)–80). Central tooth triangular, tricuspid; ectocones small; mesocone large (Fig. 10H). Lateral teeth tricuspid; endocone small; mesocone large with pointed tip; ectocone large, basal, and with pointed tip. Outer lateral teeth arranged slightly obliquely, bicuspid; endocone very small to absent; mesocone large trapezoid; ectocone basal, relatively small, and pointed tip. Latero-marginal transition starts from tooth no. 18 to 21. Marginal teeth with curved teeth, bicuspid; endocone usually larger than ectocone (Fig. 10I).

***External features.*** Living snail exhibits similar soft body morphology, pulmonary cavity and caudal structure (Fig. 7D) to that of *H. humphreysiana*. The distinct characters are the brownish to greyish body and mantle edge; right and left shell lobes absent (Fig. 1F).

##### Distribution.

Previously known only from the type locality in northern Vietnam (Maassen 2006) and Xieng Khaung, northeastern Laos (Inkhavilay et al. 2019). Recently, we have located populations from northern Thailand in Chiang Mai Province.

##### Remarks.

The shell features were carefully described in Maassen (2006). The original description of *H. esculenta* was based on seven shells and placement within *Hemiplecta* was provisional (Maassen 2006), and none of the topotypic specimens have subsequently been examined. The samples from Thailand show only minor variations from the type series, in the presence of a narrow brownish spiral band and slightly elevated spire, which we attribute to intraspecific variation. It is important to examine the genitalia of the topotypic material.

#### Hemiplecta nemorosa sp. nov.

Taxon classificationAnimaliaStylommatophoraAriophantidae

7E36A495-9869-5D46-84C4-91445104B804

http://zoobank.org/5B542C60-C447-40B9-BC1D-AD24774C4AFD

Figures 8 , 9 , 10J

##### Etymology.

The species name is derived from the Latin word “*nemoris*” meaning “full of woods or shady,” which refers to the type locality of this new species in the dense deciduous forest.

##### Type specimen.

**Figure 8.**
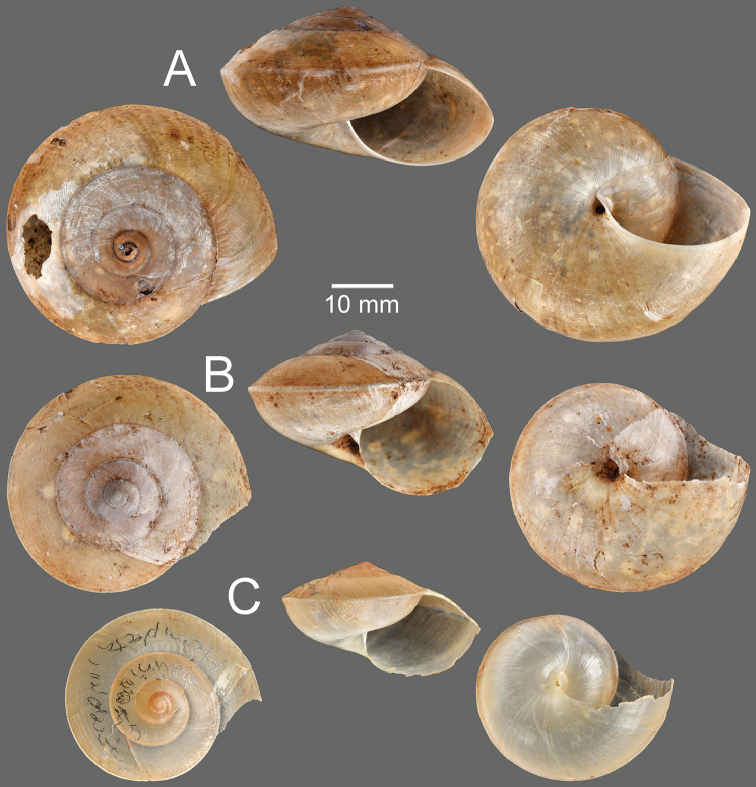
*Hemiplecta nemorosa* sp. nov. from Maehongsorn, Thailand **A** holotype CUMZ 5251 **B** paratypes CUMZ 5252, and **C** paratype CUMZ 5253 from the type locality.

***Holotype***CUMZ 5251 (height 24.6 mm, width 42.1 mm; Fig. 8A), ***paratypes***CUMZ 5252 (2 shells; Fig. 8B), CUMZ 5253 (1 adult + 1 juvenile in ethanol; Fig. 8C) all from the type locality.

##### Type locality.

Limestone outcrops with deciduous forest near road no. 1226, Pang Mapha Sub-district, Pang Mapha District, Mae Hongson Province, Thailand (19°34'10.2"N, 98°12'02.3"E).

##### Description.

Shell medium sized (height up to 15 mm, width up to 45 mm), depressed conic, thin and dextral. Whorls 5 to 6, increasing regularly, slightly convex, with very wide and shallow suture. Spire convex; apex acute; embryonic shell smooth; following whorls with thin growth lines and radial wrinkles or undulating surfaces. Periostracum thin and transparent. Shell pale brownish to yellowish. Last whorl angular with strong peripheral keel which is much reduced near aperture. Aperture not descending, widely ovate and moderately oblique; lip simple to slightly thickened in adult specimen. Columella slightly dilated; parietal callus slightly thick and translucent. Umbilicus narrowly opened, deep, and partly covered by reflected columellar lip (Fig. 8).

**Figure 9.**
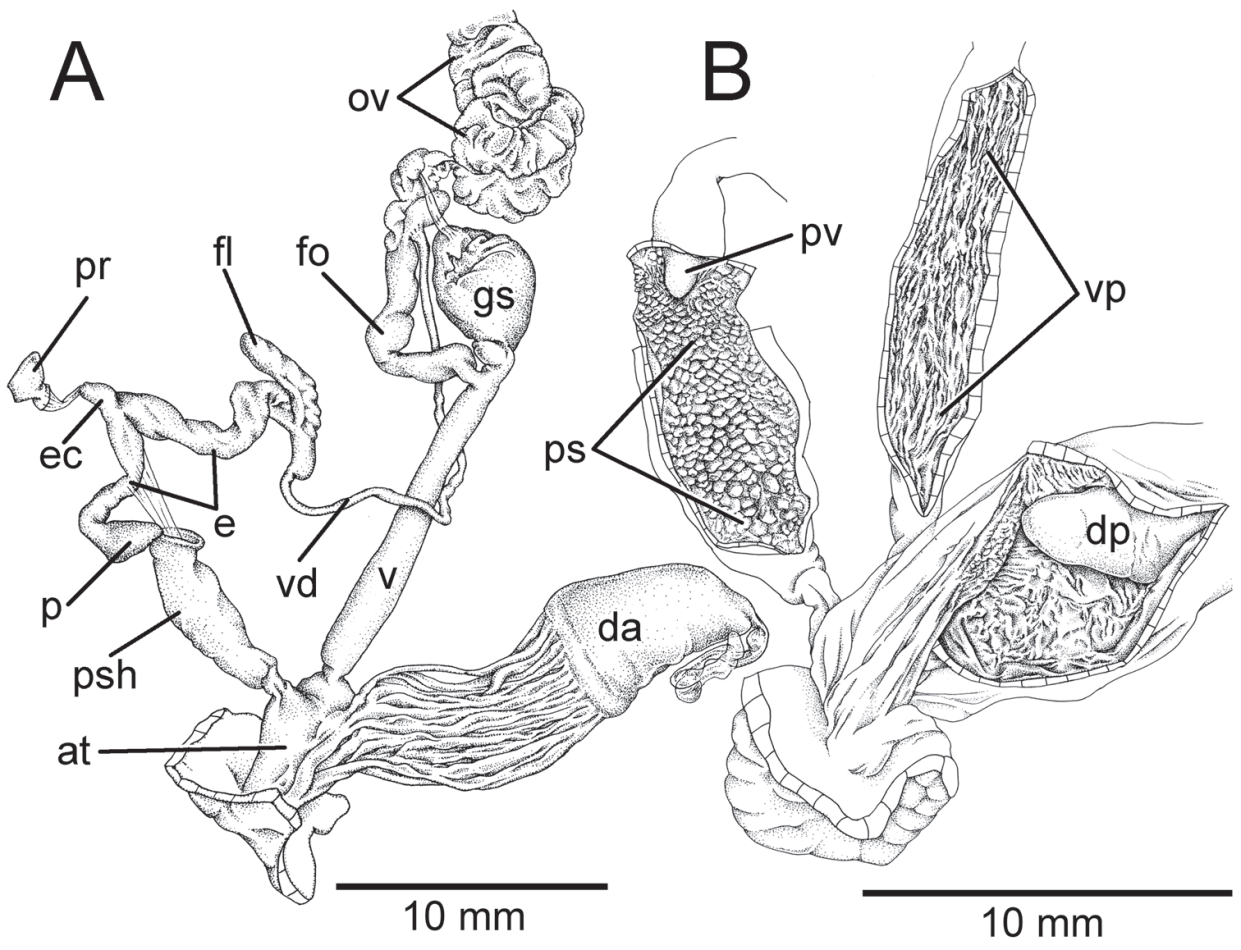
Genitalia, mantle edge structure, and caudal region of *Hemiplecta nemorosa* sp. nov., paratype CUMZ 5253 from Maehongsorn, Thailand **A** whole genital organ and **B** internal wall sculpture of penis, vagina and dart chamber.

***Genitalia.*** Atrium (at) long. Penis (p) long slender, cylindrical, and encircled by thick penial sheath (psh) extending to about half of penis length. Epiphallic caecum (ec) short, straight; penial retractor muscle (pr) thin and attached to the tip. Epiphallus (e) short, about half of penis length. Flagellum (fl) short, stout, and with thin muscle bands connected to penial sheath. Vas deferens (vd) small tube (Fig. 9A). Internal wall of penis with sculpture over entire chamber with uniform scale-like or triangular lingulate pilasters varying in size from small to large and pilasters encircling penial verge smaller than in the middle of chamber. Penial verge (pv) small, conic, and with smooth surface (Fig. 9B).

Vagina (v) long, cylindrical, about same length as penis; internal wall with thin and smooth longitudinal vaginal pilasters (vp). Dart apparatus (da) short and enlarged muscular cylinder; externally covered with thin longitudinal muscular bands around half of dart apparatus length. Internally with irregular wall, dart papilla (dp) conic and smooth. Gametolytic sac (gs) bulbous without distinct duct. Free oviduct (fo) long and encircled with thin blackish muscular tissue. Oviduct (ov) long and with lobules; prostate gland bound to oviduct. Albumen gland, hermaphroditic duct, and hermaphroditic gland missing from the examined specimen (Fig. 9A, B).

**Figure 10.**
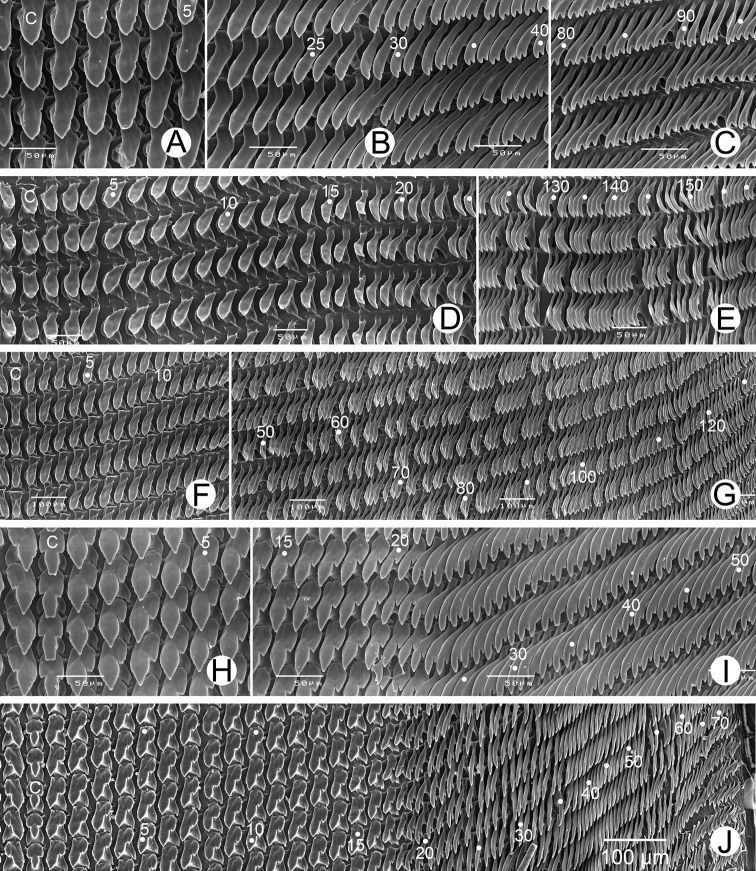
Representative SEM images of radula **A–C***Hemiplecta humphreysiana*, specimen CUMZ 4573 from Singapore **A** central and lateral teeth **B** transition from lateral teeth to marginal teeth and **C** outermost marginal teeth **D, E***Hemiplecta distincta*, specimen CUMZ 4560 from Chanthaburi, Thailand **D** central and lateral teeth and **E** outermost marginal teeth **F, G***Hemiplecta funerea*, specimen CUMZ 4575 from Nan, Thailand **F** central and lateral teeth and **G** outermost marginal teeth **H, I***Hemiplecta esculenta*, specimen CUMZ 4553 from Chiang Mai, Thailand **H** central and lateral teeth **I** transition from lateral teeth to marginal teeth **J***Hemiplecta nemorosa* sp. nov., paratype CUMZ 5253 from Maehongsorn, Thailand. Central tooth indicated by ‘C’. Numbers indicate the tooth order from lateral to marginal end.

***Radula.*** Teeth arranged in wide angled U-shape. Each row containing more than 135 teeth (+58–(16–19)–1–(16–19)–75). Central tooth symmetrical tricuspid and triangular; mesocone conic shaped and with pointed cusp; ectocones short with dull cusps located at middle of tooth height. Lateral teeth asymmetrical tricuspid; endocone nearly absent; mesocone triangular with pointed cusp; ectocone with pointed cusps and located below endocone. Marginal teeth start around tooth numbers 16 to 19, elongate and obliquely bicuspid; endocone larger than ectocone and with pointed cusp; ectocone very small. Outer marginal teeth bicuspid and shorter than inner teeth (Fig. 10J).

##### Distribution.

This new species is currently known only from the type locality in northern Thailand.

##### Remarks.

The shell morphology of this new species is similar to *H. uter* (Theobald, 1859) from Myanmar and *Falsiplecta integripedia* Schileyko & Semenyuk, 2018 from southern Vietnam. This new species, however, differs by having a shell width almost two-times larger than *H. uter*, but further comparison of anatomical characters is necessary to confirm their distinction. *Hemiplecta nemorosa* sp. nov. clearly differs from *F. integripedia* in having a well-developed dart apparatus, globular gametolytic sac, and long epiphallus and flagellum. In contrast, *F. integripedia* has no dart apparatus, a long gametolytic duct, a very short epiphallus and the vas deferens attached near the tip of the epiphallus (flagellum lacking). *Hemiplecta nemorosa* sp. nov. also differs from *H. undosa* (Blanford, 1865) by having a relatively smaller shell size, an angular last whorl with strong peripheral keel, and a narrow umbilicus. In contrast, *H. undosa* has a rounded to slightly shouldered last whorl, and a wide and deep umbilicus.

### Species list from Indochina including Peninsular Malaysia and Myanmar

This synoptic list includes all the nominal species-group names that have been attributed to *Hemiplecta* s.l. and have the type locality within the geographic area covered by mainland Indochina, Peninsular Malaysia or the southeastern part of Myanmar. All the nominal species group names are listed alphabetically where their original combinations and original publication were provided. In nearly all instances, the original literature was checked for authorship and date, page numbers of the original description and illustrations, and type locality to ensure accuracy of the entries. The usage of the nominal name, necessary references that provided descriptions or images of possible type specimens, and recent taxonomic treatment articles that placed species into the genus *Hemiplecta* are also listed. The current taxonomic status (validity or synonymy) of each taxon is provided, mainly following recent literature and this study. The depository information of the name-bearing types (holotype, lectotype, or syntype) is provided. The name-bearing types are illustrated when possible; exceptions are those recently published in Inkhavilay et al. (2019), Páll-Gergely (2019), and Sutcharit et al. (2020). However, in the cases where the name-bearing types could not be traced, topotypic or authentic reference specimens are illustrated instead for further comparison. In some instances, information about the authorship, type series, and type locality is discussed under the remarks section. The type specimens were located (preserved) in several museums, as follows:

#### Group I: Dextral species

**1 *auriettae* (Tapparone Canefri, 1889)**

Nanina (Macrochlamiys) auriettae Tapparone Canefri, 1889: 318, 319, pl. 8, figs 4–6. Type locality: Sul monte Mooleyit [Mulayit Hill, Hpa-An District, Kayin State, Myanmar].

Hemiplecta ? auriettae: Blanford and Godwin-Austen 1908: 293.

**Current taxonomic status.***Hemiplecta*. Valid species.

**Type specimens.** The type specimens could not be traced.

**Figure 11.**
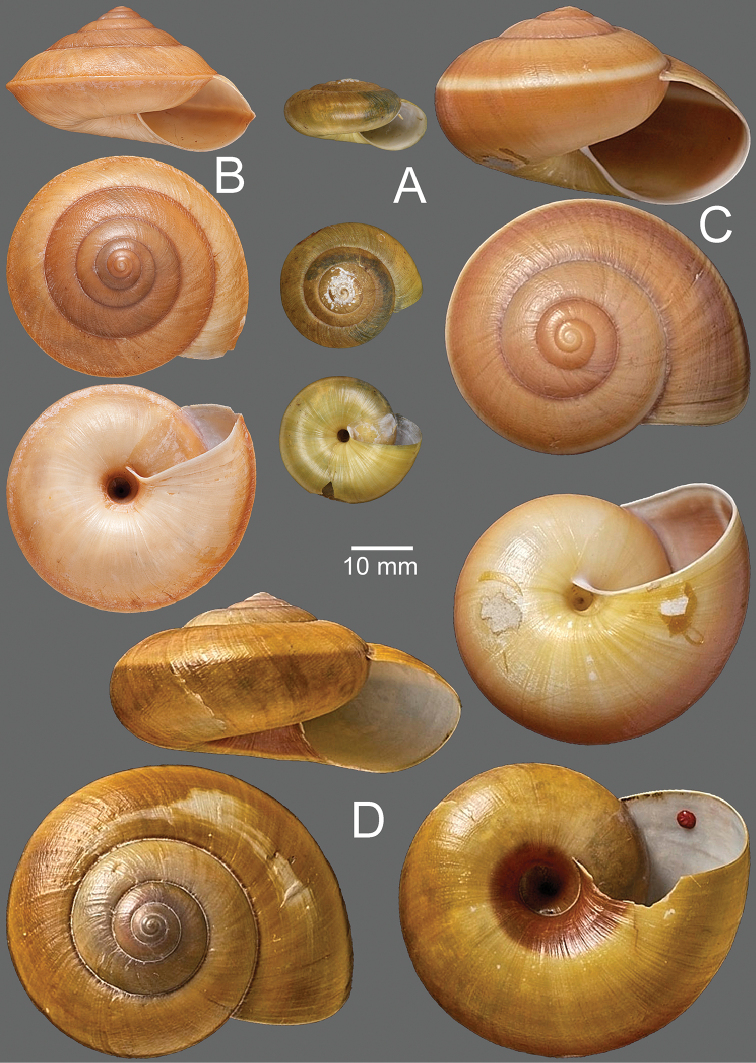
**A***Hemiplecta auriettae*, specimen NHMUK 1912.4.16.497 **B***Hemiplecta cymatium*, specimen UMZC I.104350 ex. Benson collection **C***Hemiplecta distincta*, possible syntype NHMUK 20200199 **D***Hemiplecta floweri*, syntype NHMUK 1899.3.16.1–2.

**Remarks.** The topotype specimen NHMUK 1912.4.16.497 (3 shells; Fig. 11A) from Mooley-it, Tenasserim is figured herein.

**2 *chevalierii* (Souleyet, 1842)**

*Helix chevalierii* Souleyet, 1842: 101. Type locality: Malacca [Malacca State, Malaysia].

*Helix chevalierii*: Souleyet, 1852: 503, 504, pl. 28, figs 24–26.

*Hemiplecta chevalierii*: Maassen 2001: 101.

**Current taxonomic status.***Hemiplecta*. Valid species.

**Type specimens.** The type specimens could not be traced.

**3 *cymatium* (Pfeiffer, 1856)**

*Nanina cymatium* Bens. Pfeiffer 1855: 121. [unavailable name].

*Helix cymatium* Benson. Pfeiffer, 1856b: 58, pl. 17, figs 1, 2. Type locality: Pulo Lancavi, peninsulae Malaccanae [Langkawi Islands, Kedah State, Malaysia].

*Hemiplecta cymatium*: Maassen 2001: 101, 102.

**Current taxonomic status.***Hemiplecta*. Valid species.

**Type specimens.** The type specimens could not be traced.

**Remarks.** The manuscript name “*cymatium* Bens.” was never published by Benson. It was first mentioned in the species list published by Pfeiffer (1855: 121), but without any indication to make the name available (ICZN 1999: Art. 12). Later, Pfeiffer (1856b) published this name with a description and illustration and attributed it to Benson. However, since Benson did not write the description, the authorship of this taxon should be attributed to Pfeiffer, who formally described it and made the name available.

The original description includes an illustration and one set of shell measurements. The type series of the taxa could not be traced in the UMZC and NHM collections. There are three specimens from UMZC I.104350 ex. Benson collection accompanied by a label with the taxon name but without collection locality. A specimen that closely matched the original description is figured herein (Fig. 11B).

**4 *denserugata* (Möllendorff, 1901)**

*Xestina denserugata* Möllendorff, 1901: 45, 46. Type locality: Berg Dran und Hong-gong, Süd Annam.

*Hemiplecta denserugata*: Schileyko 2011: 30.

**Current taxonomic status.***Hemiplecta*. Valid species.

**Figure 12.**
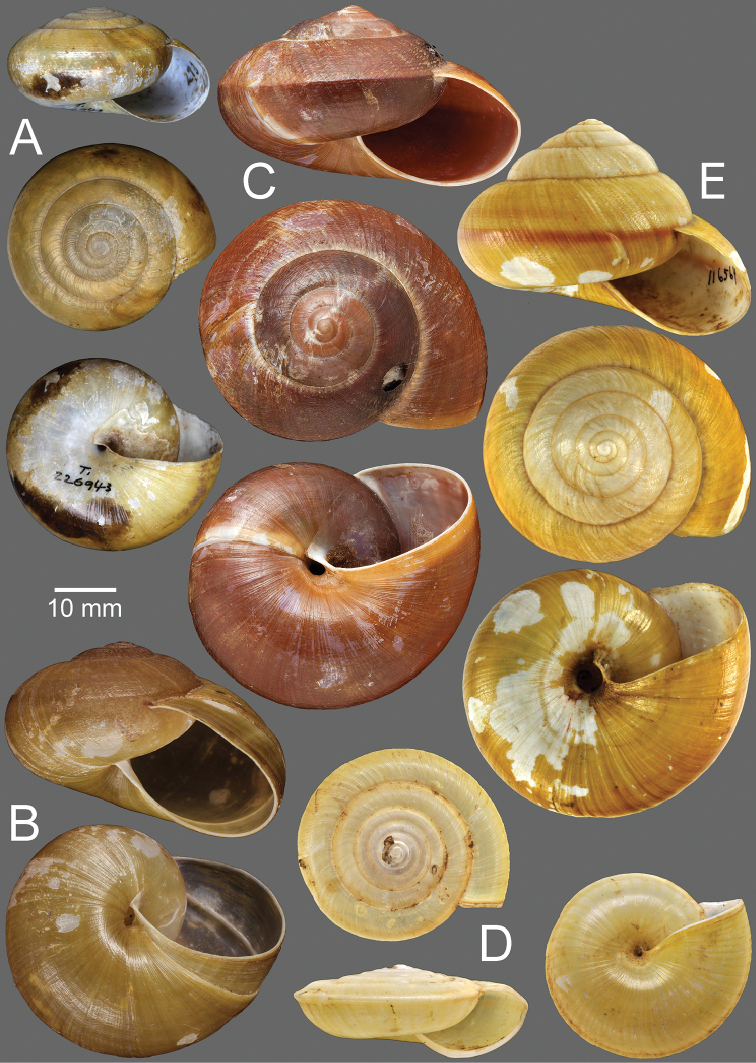
**A***Hemiplecta denserugata*, syntype SMF 226943/1 **B***Hemiplecta distincta*, holotype MNHN-IM-2000-35535 of *Hemiplecta franzhuberi* Thach, 2020 **C***Hemiplecta funerea*, lectotype NHMUK 1896.1.25.4 **D***Hemiplecta gordoniae*, specimen NHMUK 1903.7.1.309 **E***Hemiplecta humphreysiana*, syntype NMNH 116569.

**Type specimens.** Syntype SMF 226943/1 (1 shell, height 20.1 mm, width 33.2 mm; Fig. 12A) from Süd Annam, Berg Dran, 3000 ft.

**Remarks.** The number of specimens was not clearly stated and only one set of shell measurements was given in the original description. The single specimen from the type lot is illustrated herein for the first time.

**5 *distincta* (Pfeiffer, 1850)**

*Helix distincta* Pfeiffer, 1850: 69, 70. Type locality: Insulis Moluccis [possibly error or mislabeling]. Pfeiffer 1853: 346, pl. 134, figs 1, 2. Reeve 1854: *Helix* pl. 86, species 465.

Hemiplecta (Hemiplecta) distincta: Hemmen and Hemmen 2001: 44, fig. 12.

**Current taxonomic status.***Hemiplecta*. Valid species.

**Type specimens.** Possible syntype NHMUK 20200199 (3 shells; Fig. 11C) from Moluccas [possibly error or mislabeling].

**Remarks.** This species was described based on specimens from the Cuming collection. The original description did not include an illustration and only one set of shell measurements was given. Later, Pfeiffer (1853) re-published the description and figured this species based on material from the Cuming collection. The NHM collections contain a lot of three shells from the Cuming collection. The original label, not in Pfeiffer’s handwriting, states the taxon name and gives the collection locality as “Hab. Moluccas (Pfr. Zeitschr. 1850. p. 69)”, and a small printed label stating “Type?”. Additionally, the collection localities “Siam & Camboja” and “Siam & Cochin Chine (Martens)” were probably added at a later date. Therefore, we consider this lot to be possible syntypes. The specimen that closely matched the measurements in the original description and illustration in Pfeiffer (1853) is figured herein.

The museum collection and current published record with detailed geographical data of *H. distincta* are only from Indochina. Therefore, the type locality “Insulis Moluccis [Molucca Islands in eastern Indonesia]” possibly error or mislabeling.

**6 *esculenta* Maassen, 2006**

*Hemiplecta esculenta* Maassen, 2006: 17, 18, figs 10–12. Type locality: limestone area near village Hang, Pu Luong National Park, Thanh Hoa, Vietnam. Inkhavilay et al. 2019: 76, fig. 35d, e. Páll-Gergely 2019: figs 11–13.

**Current taxonomic status.***Hemiplecta*. Valid species.

**Type specimens.** Holotype RMNH 99424 (see Inkhavilay et al. 2019: fig. 35d), paratype RMNH 99425 (1 shell).

**Remarks.** The type specimen was recently illustrated in Inkhavilay et al. (2019) and Páll-Gergely (2019).

**7 *floweri* Smith, 1899**

*Hemiplecta floweri* Smith, 1899: 284, 285, text figures. Type locality: Maxwell’s Hill, Larut, Perak [Bukit Larut, Taiping, Perak State, Malaysia]. Maassen 2001: 103.

**Current taxonomic status.***Hemiplecta*. Valid species.

**Type specimens.** Syntype NHMUK 1899.3.16.1–2 (2 shells; Fig. 11D) from Malay Peninsula.

**Remarks.** The original description included illustrations and one set of shell measurements. However, the species description was not explicitly based on one specimen. There are two shells in the NHM type lot with an original label stating “Types”, subsequently changed to read “holotype red spot”. The shell that matched the measurements given in the original description and that has a red spot in the aperture is figured herein.

**8 *franzhuberi* Thach, 2020**

*Hemiplecta franzhuberi* Thach, 2020: 38, figs 442–444. Type locality: Thakhek, Laos.

**Current taxonomic status.***Hemiplecta*. Synonym of *Hemiplecta distincta*.

**Type specimens.** Holotype MNHN-IM-2000-35535 (Fig. 12B) from Thakhek, Savannakhet, Laos.

**Remarks.** This species seems to be described based on a single specimen and the author refers to the diagnosed character “two broad shallow spiral grooves situated near the periphery of body whorl at dorsal side”. However, using just one character without any further independent diagnostic characters could raise doubt about the taxonomic status. The single shell may reflect an abnormality during the growth stage and, apart from this trait, all the other shell characters all are within the range of morphological variations seen within *H. distincta*. Therefore, we consider *H. franzhuberi* as a junior synonym of the more common and widespread *H. distincta*.

It would be very useful if this new species were compared with sympatric or geographically proximate species (i.e. *H. distincta* or *H. pluto*) instead of the distant species *H. abbasi* Maassen, 2009 from Sumatra (Maassen 2009).

**9 *funerea* (Smith, 1896)**

Nanina distincta var. funerea Smith, 1896: 128. Type Locality: Vanbu, Tonkin [Van Ban District, Lao Cai Province, Vietnam]. Fischer and Dautzenberg 1904: 393.

*Hemiplecta funerea*: Inkhavilay et al. 2019: 76, 77 [not figs 35f, 36a].

**Current taxonomic status.***Hemiplecta*. Valid species.

**Type specimens.** Lectotype (design. n.) NHMUK 1896.1.25.4 (1 shell; Fig. 12C) designated from Vanbu, Tonkin.

**Remarks.** The species description is clearly based on more than one specimen. The original description does not include an illustration, and measurements of the largest specimen are given. The NHM collection contains a lot of a single specimen with a label stating “var. funerea”. This specimen matched well with the original description and is here designated as the lectotype to stabilize the name.

Inkhavilay et al. (2019: fig. 35f) state this specimen is the syntype of “var. funerea” but wrongly apply the images of “var. pallidior” instead.

**10 *gordoniae* (Benson, 1863)**

*Helix gordoniae* Benson, 1863: 87. Type locality: Birmanica prope Moulmein [Mawlamyine Township, Mawlamyine District, Mon State, Myanmar]. Hanley and Theobald 1870: 13, pl. 27, figs 1, 2.

Hemiplecta ? gordoniae: Blanford and Godwin-Austen 1908: 293.

**Current taxonomic status.***Hemiplecta*. Valid species.

**Type specimens.** The type specimens could not be traced from the Benson collection.

**Remarks.** The topotype specimen from Godwin-Austen collection NHMUK 1903.7.1.309 (1 shell; Fig. 12D) with collection locality from Needoung Thoung, Ataran valley, Tenasserim is figured herein.

**11 *huberi* Thach, 2017**

*Hemiplecta huberi* Thach, 2017: 33, figs 389–391. Type locality: Thakhek, Khammouane Province, Central Laos. Inkhavilay et al. 2019: 77, fig. 36b. Páll-Gergely et al. 2020: 46.

**Current taxonomic status.***Hemiplecta*. Synonym of *Hemiplecta pluto*.

**Type specimens.** Holotype MNHN-IM-2000-33196 (see Inkhavilay et al. 2019: fig. 36b).

**Remarks.**Páll-Gergely et al. (2020) attributed the diagnostic character of this name as a morphological variation of the widely distributed *H. pluto*, so this is treated as a junior synonym.

**12 *huberi* Thach, 2017**

*Helminthoglypta huberi* Thach, 2017: 54, figs 747–749 [non *Hemiplecta huberi* Thach, 2017: 33, figs 389–391]. Type locality: Thakhek, Khammouane Province, Central Laos.

**Remarks.** See under *Hemiplecta lanxangnica* Inkhavilay & Panha, 2019

**13 *humphreysiana* (Lea, 1840)**

*Helix humphreysiana* Lea, 1840: 175. Type locality: Pondicherry and Singapore. Lea 1841: 463, 464, pl. 12, fig. 16.

*Hemiplecta humphreysiana*: Godwin-Austen 1898: 74–76, pl. 80, fig. 6, 6b; pl. 81, fig. 1, 1e. Benthem Jutting 1959: 148–150.

**Current taxonomic status.***Hemiplecta*. Valid species.

**Type specimens.** Syntype NMNH 116569 (1 shell; Fig. 12E) from Pondicherry.

**Remarks.** This species was clearly described based on more than one specimen. The author’s description clearly indicates that the type series was from two collection localities: “Pondicherry” received from Mr. Humphreys and “Singapore” received from Mr. Balastire. Later, Lea (1841) re-described the species and illustrated a single specimen. The Smithsonian collections contain a lot of a single shell from the Lea collection as from “Pondicherry”. This specimen matched well with the illustration and the measurements given in the original description.

The records of this species from “Pondicherry” [the historical name probably referred to the cities on the east coast of India] have never been verified. Currently, the genus *Hemiplecta* are distributed from Southeast Asia to Southeast Asia and New Guinea, except one species recorded from the Maldives (Schileyko 2002). Therefore, “Pondicherry” is probably an erroneous record (see also Godwin-Austen 1898: 74), and “Singapore” is possibly the correct type locality of this species.

**14 *jensi* Páll-Gergely, 2019**

*Hemiplecta jensi* Páll-Gergely, 2019: 86–88, figs 1–6. Type locality: Vietnam, Thanh Hoa Province, Pu Luong N.R., surroundings of Village Am.

**Current taxonomic status.***Hemiplecta*. Valid species.

**Type specimens.** Holotype SMF 353501 (see Páll-Gergely 2019: figs 1–4), from Vietnam, Thanh Hoa Province, Pu Luong N.R., surroundings of Village Am.

**15 *khamducensis* (Thach & Huber, 2018)**

*Camaena khamducensis* Thach & Huber in Thach, 2018: 67, figs 886–888. Type locality: Kham Duc area, Phuoc Son, District, Quang Nam Province, Central Vietnam.

*Hemiplecta khamducensis*: Páll-Gergely et al. 2020: 46.

**Current taxonomic status.***Hemiplecta*. Valid species.

**Type specimens.** Holotype FMNH 386292.

**Remarks.** This nominal species was transferred to the genus *Hemiplecta* by Páll-Gergely et al. (2020); we agree with their decision. An image of a living specimen in the original description (Thach 2018: fig. 888) shows an aulacopod type of pedal groove, whereas Camaenidae has a holopod type of pedal groove (see Solem 1959: fig. 2). The assignment of this species to the genus *Hemiplecta* is most likely, due to the helicarionoid snails having a relatively large shell size, simple apertural lip (slightly thickened), and narrow umbilicus (Schileyko 2002). Anatomical examination will help elucidate the appropriate generic position of this species.

Thach (2018) mentioned depositing the holotype at the Field Museum of Natural History, Chicago. However, the holotype has not arrived at the FMNH collection (Jochen Gerber, personal communication on October 2020).

**16 *khamducensis* Thach & Huber, 2000**

*Hemiplecta khamducensis* Thach & Huber in Thach, 2020: 38, 39, figs 434–437. Type locality: Kham Duc, Phuoc Son District, Quang Nam Province, Central Vietnam.

**Current taxonomic status.***Hemiplecta*. Junior homonym and junior synonym of *Hemiplecta khamducensis* (Thach & Huber, 2018).

**Type specimens.** Holotype NHMUK 20200208.

**Remarks.** This species was originally proposed as a junior secondary homonym from the same locality as the senior homonym. Basically, this junior homonym agrees well in all diagnostic shell characters of a red-brown shell, strong peripheral keel, and shell shape that all lie within the range of variation of the present species. This species is synonymized with *H. khamducensis* (Thach & Huber, 2018) herein, and, therefore, the replacement name is not necessary at present.

Thach (2020) stated that the holotype was deposited at the Natural History Museum in London. However, the holotype has not arrived in the NHM collection (Jonathan Ablett, personal communication on May 2021).

**17 *lanxangnica* Inkhavilay & Panha, 2019**

*Helminthoglypta huberi* Thach, 2017: 54, figs 747–749 [non *Hemiplecta huberi*Thach 2017: 33, figs 389–391]. Type locality: Thakhek, Khammouane Province, Central Laos.

*Hemiplecta lanxangnica* Inkhavilay & Panha in Inkhavilay et al., 2019: 77, 78, fig. 36c, d. [new replacement name]. Páll-Gergely et al. 2020: 46.

**Current taxonomic status.***Hemiplecta*. Valid species.

**Type specimens.** Holotype RMNH 5006710 from Thakhek, Khammouane Province, Central Laos. Paratype MNHN-IM-2000-33215 (1 shell; see Inkhavilay et al. 2019: fig. 36c).

**18 *laotica* (Möllendorff, 1899)**

Bensonia (Oxytes) laotica Modendorff, 1899: 165. Type locality: Oberer Mekong im Lande der Laos [upper Mekong in Laos].

*Ariophanta laotica*: Inkhavilay et al. 2019: 75, fig. 34d, e.

*Hemiplecta laotica*: Páll-Gergely 2019: 87, figs 6–10. Páll-Gergely et al. 2019: 605, fig. 7k–n.

**Current taxonomic status.***Hemiplecta*. Valid species.

**Type specimens.** Syntype SMF 226681 (1 shell; see Inkhavilay et al. 2019: fig. 34d), SMF 226682 (3 shells) from Laos.

**Remarks.** The type specimen was recently illustrated in Inkhavilay et al. (2019: fig. 34d).

**19 *malaouyi* (Morgan, 1885)**

*Xesta malaouyi* Morgan, 1885a: 374, 375, pl. 5, fig. 4. Type locality: Mont Kerbou, à 1800 mètre environ ďaltitude [1800 m altitude, Gunung Korbu, Hulu Kinta, Perak State, Malaysia].

*Hemiplecta malaouyi*: Maassen 2001: 103.

**Current taxonomic status.***Hemiplecta*. Valid species.

**Figure 13.**
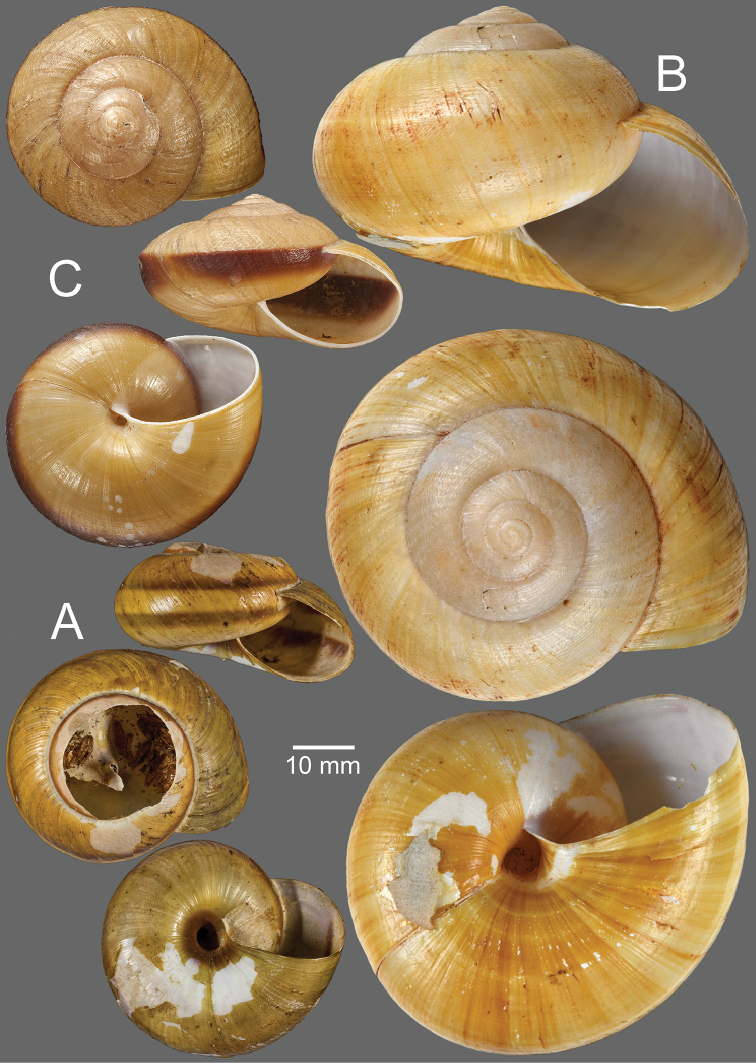
**A***Hemiplecta malaouyi*, syntype MNHN-IM-2000-34170 **B***Hemiplecta distincta*, syntype NHMUK 20150065 of *Helix neptunus* Pfeiffer, 1861 **C***Hemiplecta funerea*, syntype NHMUK 1896.1.25.5 of Nanina distincta var. pallidior Smith, 1896.

**Type specimens.** Syntype MNHN-IM-2000-34170 (1 shell; Fig. 13A) from Perak, Mont Kerbou.

**Remarks.** Only one specimen in the syntype lot and the spire was broken after the original description.

**20 *nemorosa* sp. nov.**

**Remarks.** The species is described herein (see systematic part).

**21 *neptunus* Pfeiffer, 1861**

*Helix neptunus* Pfeiffer, 1861a: 190. Type locality: Siam [Thailand]. Pfeiffer 1861b: 176, 177. pl. 48, figs 1, 2.

Hemiplecta (Hemiplecta) neptunus: Hemmen and Hemmen 2001: 44.

**Current taxonomic status.***Hemiplecta*. Synonym of *Hemiplecta distincta*.

**Type specimens.** Syntype NHMUK 20150065 (2 shells; Fig. 13B) from Siam.

**Remarks.** This name was described based on specimens from the Cuming ex. Mouhot collection. The original description did not include illustrations, and only one set of measurements was given. Later, Pfeiffer (1861b) re-described and illustrated a single specimen from the Cuming collection. There are two specimens from the Cuming collection in the NHM type lot with an original label in Pfeiffer’s handwriting stating the species name, collection locality and “*pernobilis* Fer. Var.? and pl.74, f. 4.”. The specimen that corresponded to the shell measurements in the original description and illustration in Pfeiffer (1861b) is figured herein.

**22 *pallidior* (Smith, 1896)**

Nanina distincta var. pallidior Smith, 1896: 128. Type locality: Vanbu, Tonkin [Van Ban District, Lao Cai Province, Vietnam]. Fischer and Dautzenberg 1904: 393. Inkhavilay et al. 2019: fig. 35f.

**Current taxonomic status.***Hemiplecta*. Synonym of *Hemiplecta funerea*.

**Type specimens.** Syntype NHMUK 1896.1.25.5 (1 shell; Fig. 13C) from Vanbu, Tonkin.

**Remarks.** The image of Inkhavilay et al. (2019: fig. 35f) under the name *H. funerea* is the syntype of Nanina distincta var. pallidior Smith, 1896.

**23 *pernobilis* (Férussac, 1821)**

*Helix pernobilis* Férussac, 1821: 39, no. 182. Type locality: Poulo-Condor [Con Dao Island, South Vietnam].

*Koratia distincta pernobilis*: Schileyko 2011: 30

*Koratia pernobilis*: Schileyko 2015: 15–18, fig. 1.

**Current taxonomic status.***Hemiplecta*. Valid species.

**Type specimens.** The type specimens could not be traced.

**24 *pharangensis* (Möllendorff, 1901)**

*Xestina pharangensis* Möllendorff, 1901: 46. Type locality: Pharang, Süd Annam [Phan Rang, Ninh Thuan Province, south Vietnam].

*Hemiplecta pharangensis*: Schileyko 2011: 30.

**Current taxonomic status.***Hemiplecta*. Valid species.

**Figure 14.**
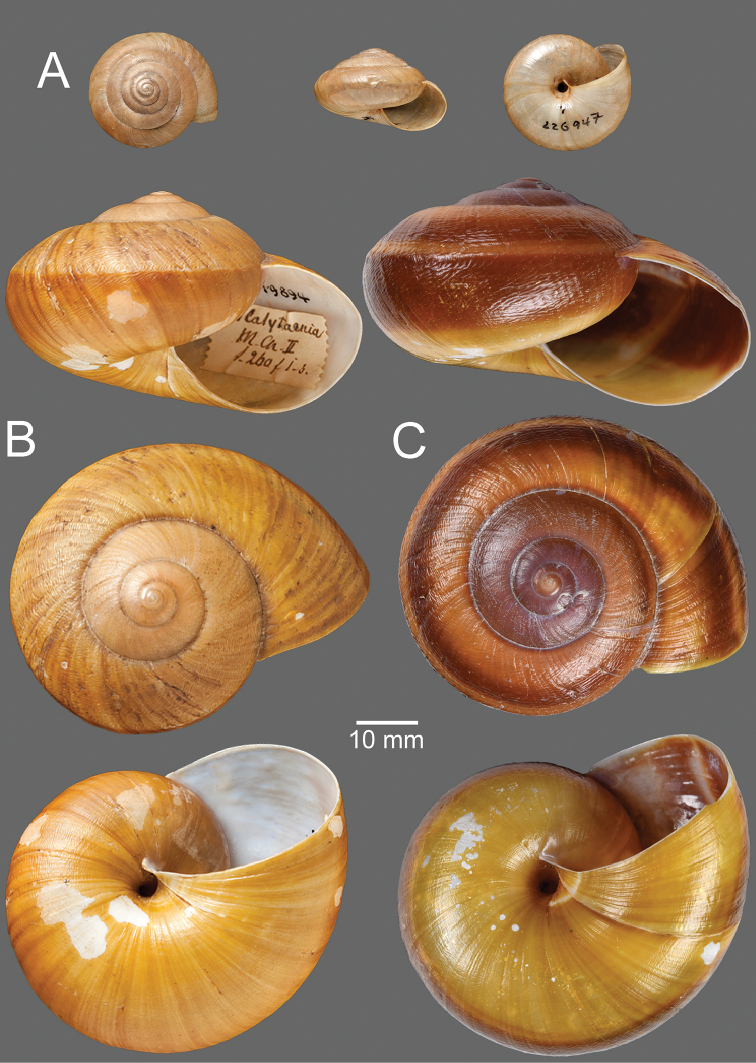
**A***Hemiplecta pharangensis*, syntype SMF 226947/1 **B***Hemiplecta platytaenia*, syntype SMF 149894 **C***Hemiplecta pluto*, lectotype NHMUK 20200200.

**Type specimens.** Holotype SMF 226947/1 (height 15.1 mm, width 22.5 mm; Fig. 14A) from Pharang, Süd-Annam.

**Remarks.** The number of specimens was not clearly stated, and only one set of shell measurements was given in the original description. The single specimen from the type lot is illustrated herein for the first time.

**25 *platytaenia* Möllendorff, 1900**

*Hemiplecta platytaenia* Möllendorff, 1900: 121. Type locality: Touranne [Da Nang, Vietnam]. Schileyko 2011: 30.

**Current taxonomic status.***Hemiplecta*. Valid species.

**Type specimens.** Syntype SMF 149894 (1 shell, height 41.2 mm, width 60.5 mm; Fig. 14B) from Annam: Touranne.

**Remarks.** The number of specimens was not clearly stated, and only one set of shell measurements was given in the original description. The single specimen from the type lot is illustrated herein for the first time. Möllendorff (1900: 121) also stated that this species differed from *H. neptunus* (=*H. distincta*) in having a flatter shape, with a weak keel, and a spiral band on periphery.

**26 *pluto* (Pfeiffer, 1863)**

*Helix pluto* Pfeiffer, 1863a [1862]: 268, 269. Type locality: Lao Mountains, Camboja [Luang Prabang, Laos]. Pfeiffer 1863b: 210, pl. 55, figs 8, 9.

Nanina (Hemiplecta) pluto: Kobelt 1900: 987, pl. 256, figs 1, 2.

*Hemiplecta pluto*: Schileyko 2011: 30. Inkhavilay et al. 2019:78, figs 36e, f, 56d. Páll-Gergely et al. 2020: 46.

**Current taxonomic status.***Hemiplecta*. Valid species.

**Type specimens.** Lectotype (design. n.) NHMUK 2020200 (1 shell; Fig. 14C) from Lao Mountains, Camboja.

**Remarks.**Pfeiffer (1863a) stated that this species was described based on specimens from the Cuming collection. The original description did not include an illustration, and only one set of shell measurements was given. There are two specimens in the mixed type-lot of different species. The specimen that had an original label in Pfeiffer’s handwriting states “*H. pluto* Pfr.” and the collection locality “Lao Mountains, Camboja”. The specimen that matched the description and shell measurements given in the original description, and the illustration in Pfeiffer (1863b: pl. 55, figs 8, 9) is here designated as the lectotype to stabilize the name.

The other shell from the same collection lot with label stating “var. neptunus young” was identified as *H. distincta*. This specimen is not part of the type series and, therefore, excluded from this designation.

Pfeiffer (1863a) described this species based on the specimen collected by H. Mouhot in the Cuming collection, and “Lao Mountains, Camboja” is the type locality. We have seen specimens with more precise geographical location from the Khammouan Province to Luang Prabang Province, Laos (Inkhavilay et al. 2019).

**27 *sakaya* (Morgan, 1885)**

*Oxytes sakaya* Morgan, 1885a: 380, 381, pl. 6, fig. 1. Type locality: Mont Kerbou, à 1200 mètre environ ďaltitude [1200 m altitude, Gunung Korbu, Hulu Kinta, Perak State, Malaysia].

*Hemiplecta sakaya*: Laidlaw, 1932a: 89. Maassen, 2001: 101.

**Current taxonomic status.***Hemiplecta*. Synonym of *Hemiplecta cymatium*.

**Figure 15.**
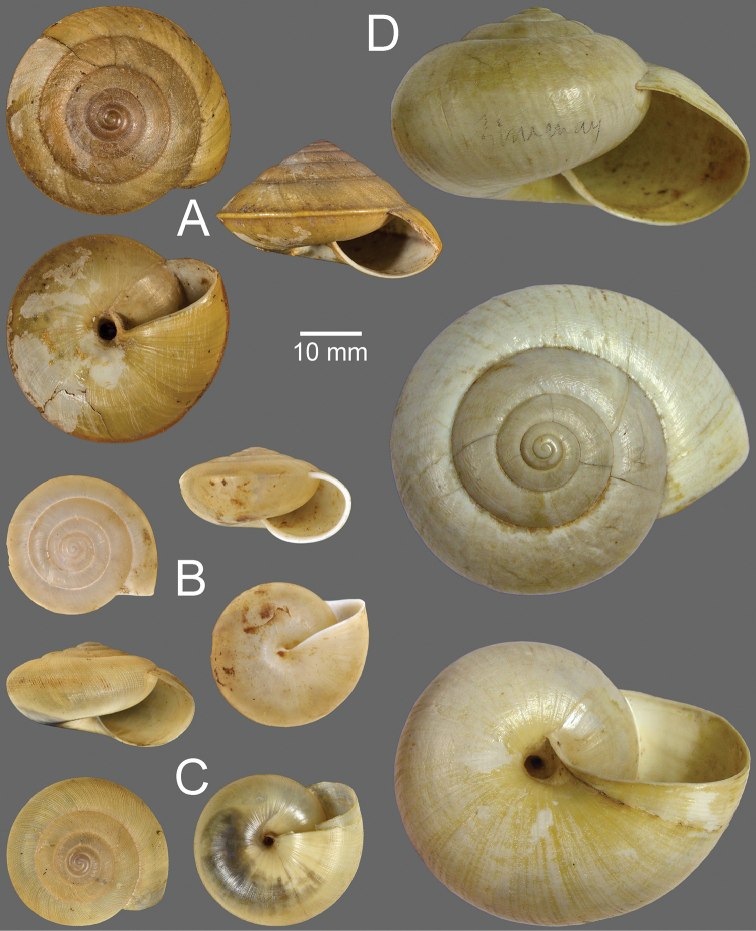
**A***Hemiplecta sakaya*, syntype MNHN-IM-2000-34169 **B***Hemiplecta textrina*, specimen NHMUK 1906.1.1.389 **C***Hemiplecta theodori*, specimen NHMUK 1888.12.4.1517 **D***Hemiplecta distincta*, syntype NHMUK 1888.12.4.2007 of *Hemiplecta zimmayensis* Godwin-Austen, 1888.

**Type specimens.** Syntype MNHN-IM-2000-34169 (2 shells; Fig. 15A) from Presqúîle de Malacca, mont Kerbou.

**Remarks.**Laidlaw (1932a) seems to be the first to synonymize this species with *H. cymatium*, and this treatment has been followed by Maassen (2001) until recently. The type specimen ex. de Morgan collection is figured herein.

**28 *textrina* (Benson, 1856)**

*Helix textrina* Benson, 1856: 252. Type locality: ad Thyet Myo [Thayet District, Magway Region, Myanmar]. Pfeiffer 1860: 131, pl. 36, figs 5–7. Hanley and Theobald 1872: 24, pl. 52, figs 2, 5.

Hemiplecta ? textrina: Blanford and Godwin-Austen 1908: 292.

**Current taxonomic status.***Hemiplecta*. Valid species.

**Type specimens.** The type specimens could not be traced.

**Remarks.** The specimen from the Blanford collection NHMUK 1906.1.1.389 from Bassein, Pegu is figured herein (Fig. 15B).

**29 *theodori* (Philippi, 1846)**

*Helix theodori* Philippi, 1846: 191, 192. Type locality: Prope Mergui Indiae orientalis [Myeik Township, Myeik District, Tanintharyi Region, Myanmar].

*Hemiplecta theodori*: Blanford and Godwin-Austen 1908: 292, 293.

**Current taxonomic status.***Hemiplecta*. Valid species.

**Type specimens.** The type specimens could not be traced.

**Remarks.** The topotype specimen NHMUK 1888.12.4.1517 (1 shell; Fig. 15C) from Mergui is figured herein.

**30 *undosa* (Blanford, 1865)**

Nanina (Hemiplecta) undosa Blanford, 1865: 68. Type locality: Shan Hills, east of Ava [Shan Hills in Kyaukse District, Mandalay Region, Myanmar].

*Helix undosa* var. Hanley & Theobald, 1874: 45, pl. 111, figs 2, 3.

*Hemiplecta undosa*: Blanford and Godwin-Austen 1908: 291, 292.

**Current taxonomic status.***Hemiplecta*. Valid species.

**Figure 16.**
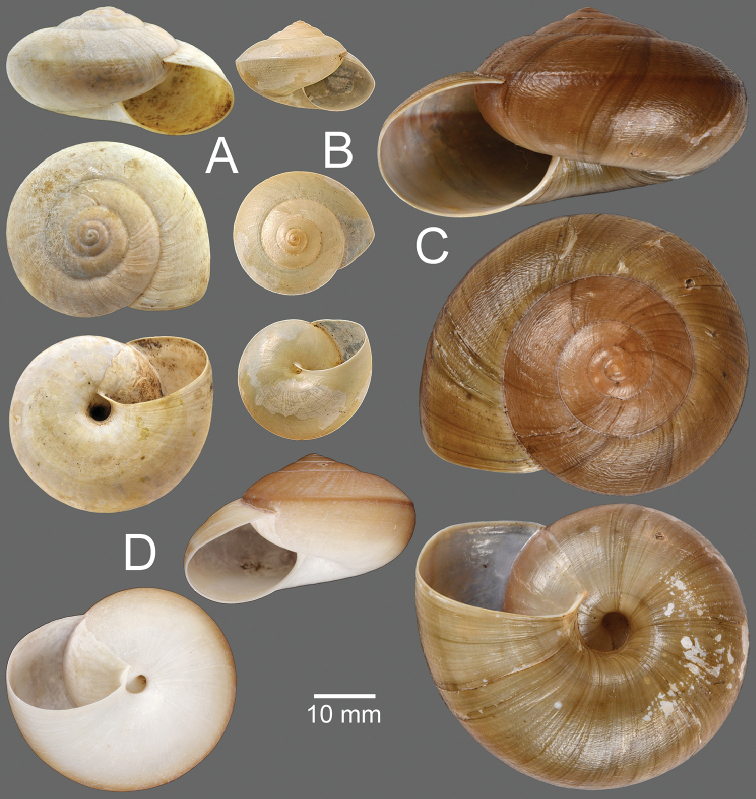
**A***Hemiplecta undosa*, probable syntype NHMUK 20200201 **B***Hemiplecta uter*, holotype NMHUK 1888.12.4.1487 **C***Hemiplecta salangana*, syntype NHMUK 1904.5.26.18–19 of Hemiplecta salangana var. martensi Collinge, 1903 **D***Hemiplecta salangana*, holotype MNHN-IM-2000-35533 of *Ariophanta trangensis* Thach & Huber, 2020.

**Type specimens.** Probable syntype NHMUK 20200201 (2 shells; Fig. 16A) from Ava, Hills east of Shan State.

**Remarks.** The original description did not include an illustration, and only one set of shell measurements was given. The author stated, “All the specimens”, implying that this description was based on more than one specimen. Blanford (1865) also mentioned “? horny when fresh” and “…dead and bleached” in the original description. The NHM collection contains a lot of two bleached shells from the Godwin-Austen ex. Blanford collection with an original label stating the species name; however, this is probably not in Blanford’s handwriting. Therefore, we consider this lot to be a probable syntype. However, the specimen that matched well with the original description and shell dimensions is figured herein.

**31 *uter* (Theobald, 1859)**

*Helix uter* Theobald, 1859: 305. Type locality: Maulmein [Mawlamyine Township, Mawlamyine District, Mon State, Myanmar]. Hanley and Theobald 1872: 27, pl. 58, figs 7, 8.

*Hemiplecta uter*: Blanford and Godwin-Austen 1908: 291.

**Current taxonomic status.***Hemiplecta*. Valid species.

**Type specimens.** Holotype NMHUK 1888.12.4.1487 (height 16.3 mm, width 26.5 mm; Fig. 16B) from Moulmein.

**Remarks.** The original description clearly states that this taxon was described based on only one specimen collected by W.S. Atkinson (Theobald 1859). The NHM registration records show that a specimen was purchased from W. Theobald with the label stating “type” and locality given as “Moulmein”. Therefore, this single specimen is recognized as the holotype fixed by monotypy (ICZN 1999: Art. 73.1.2).

**32 *zimmayensis* Godwin-Austen, 1888**

Hemiplecta ? zimmayensis Godwin-Austen, 1888c: 241, 242. Type locality: Zimmay, Siam territory [Chiang Mai Province, Thailand].

Hemiplecta (Hemiplecta) zimmayensis: Hemmen and Hemmen 2001: 44.

**Current taxonomic status.***Hemiplecta*. Synonym of *Hemiplecta distincta*.

**Type specimens.** Syntypes NHMUK 1888.12.4.2007 (1 shell; Fig. 15D) from Zimmay territory, Siam; NHMUK 1903.7.1.2108 (1 shell) from Siam.

**Remarks.** The original description did not contain any illustrations, and only one set of measurements was given. Godwin-Austen stated that the type series was from his own and Theobald’s collections. The NHM collection contains two lots that are considered to constitute the type series. Lot NHMUK 1903.7.1.2108 consists of a single specimen from the Godwin-Austen ex. Theobald collection and has original labels giving the species name “*zimmayensis*” and type collection locality “Siam”. The other lot consists of a single shell, NHMUK 1888.12.4.2007, and the NHM registration book shows that this specimen lot was purchased from W. Theobald, and with an original label stating the species name “*H. Zimmayensis* G.A.” and type collection locality “Zimmay territory (Siam)”. This specimen (NHMUK 1903.7.1.2108) from the Godwin-Austen collection is figured herein.

#### Group II: Sinistral species

**33 *lahatensis* (Morgan, 1885)**

*Helix lahatensis* Morgan, 1885b: 69. Type locality: dans la forêt Lahat et Ipoli [Lahat, Ipoh, Perak State, Malaysia].

*Ariophanta lahatensis*: Morgan 1885a: 382, pl. 6, fig. 4.

*Dyakia lahatensis*: Laidlaw 1931: 193.

*Hemiplecta lahatensis*: Sutcharit et al. 2021: 206, 207, figs 3e, 4a.

**Current taxonomic status.***Hemiplecta*. Valid species.

**Type specimens.** Syntype MNHN-IM-2000-22834 (3 shells; see Sutcharit et al. 2021: fig. 3e) from Royaume de Pérak, vallée de Kinta.

**Remarks.** The type specimen of this nominal species seems to be based on the immature shell, and the genital organ was not examined. However, the molecular phylogeny based on the juvenile specimens from approximate type locality strongly suggests it is a member of the genus *Hemiplecta* (Sutcharit et al. 2021).

**34 *ligorica* Sutcharit & Panha, 2021**

*Hemiplecta ligorica* Sutcharit & Panha in Sutcharit et al. 2021: 208, 209, figs 4d, e, 5e, f, 6f. Type locality: The limestone hills at Tam Khao Lek, Nop Phitam District, Nakhon Si Thammarat Province, Thailand.

**Current taxonomic status.***Hemiplecta*. Valid species.

**Type specimens.** Holotype CUMZ 5093/1.

**Remarks.** The species was recently described. Shell, genitalia, and DNA phylogeny confirm their generic status within the genus *Hemiplecta*.

**35 *martensi* (Collinge, 1903)**

Hemiplecta salangana var. martensi Collinge, 1903: 209. Type locality: Bukit Bersa [area in Khok Pho District, Pattani Province, Thailand].

**Current taxonomic status.***Hemiplecta*. Synonym of *Hemiplecta salangana*.

**Type specimens.** Syntype NHMUK 1904.5.26.18–19 (2 shells; Fig. 16C) ex. Annandale and Robinson collection from Bukit Bersa.

**Remarks.** The original description does not include any illustration, and the author clearly stated that measurements given were based on two specimens. There is a specimen lot in the NHM ex. Annandale and Robinson collection consisting of two shells; one of these has a malformed shell form as stated in the original description. These two shells are considered as the syntypes and figured herein for the first time.

A recent phylogenetic study has recognized this taxon as a junior synonym of *H. salangana*, a widespread species in the southern peninsula of Thailand and northern Peninsular Malaysia (Sutcharit et al. 2021).

**36 *retrorsa* (Gould, 1843)**

*Helix retrorsa* Gould, 1843: 139. Type locality: Tavoy, British Burma [Dawei District, Tanintharyi Region, Myanmar]. Johnson 1964: 140, pl. 38, fig. 10.

Helix (Caracolla) retrorsa: Gould 1844: 455, pl. 24, fig. 5.

*Dyakia retrorsa*: Blanford and Godwin-Austen 1907: 300. Laidlaw, 1931: 191.

*Hemiplecta retrorsa*: Sutcharit et al. 2021: 200–205, figs 3a, b, 5a, b, 6a–c.

**Current taxonomic status.***Hemiplecta*. Valid species.

**Type specimens.** Lectotype (designated by Johnson (1964)) MCZ 169330 (1 shell, see Sutcharit et al. 2020, fig. 3a) from Tavoy, British Burma. Paralectotype MCZ 169331 (1 shell), USNM 611233 (1 shell), MCZ 220663 (1 shell).

**Remarks.** The type specimen was recently figured, and recent systematic revision based on both genitalia morphology and DNA phylogeny confirm their generic status within the genus *Hemiplecta* (Sutcharit et al. 2021).

**37 *salangana* (Martens, 1883)**

*Nanina salangana* Martens, 1883: 134–136, pl. 25, figs 8–12. Type locality: insulam Salanga (Junk Ceilon) ad oram occidentalem peninsulae Malaccanae [Phuket Province, Thailand and Peninsular Malaysia]. Sutcharit et al. 2012: 280, fig. 2.

*Dyakia salangana*: Laidlaw 1931: 191. Berry 1963: 14, pl. 9, fig. 61.

*Hemiplecta salangana*: Sutcharit et al. 2021: 205, 206, fig. 3c, d.

**Current taxonomic status.***Hemiplecta*. Valid species.

**Type specimens.** Syntypes ZMB/Moll 32578 (1 adult +1 juvenile, see Sutcharit et al. 2020: fig. 3c) from Salanga; ZMB/Moll 57522 (1 shell) from Salanga Hinterindien.

**Remarks.** The type specimen was recently published.

**38 *thailandica* Sutcharit & Panha, 2021**

*Hemiplecta thailandica* Sutcharit & Panha in Sutcharit et al. 2021: 207, 208, figs 4b, c, 5c, d, 6d, e. Type locality: Primary evergreen forest at Khao Soidao, Soidao District, Chanthaburi Province, Thailand.

**Current taxonomic status.***Hemiplecta*. Valid species.

**Type specimens.** Holotype CUMZ 5095/1.

**Remarks.** The species was recently described. Shell, genitalia, and DNA phylogeny analyses confirm their generic status within the genus *Hemiplecta*.

**39 *trangensis* Thach & Huber, 2020**

*Ariophanta trangensis* Thach & Huber in Thach, 2020: 36, 37, figs 446–447. Type locality: Suburb of Trang City, Trang Province, Thailand.

*Hemiplecta salangana*: Sutcharit et al. 2021: 206.

**Current taxonomic status.***Hemiplecta*. Synonym of *Hemiplecta salangana*.

**Type specimens.** Holotype MNHN-IM-2000-35533 (Fig. 16D).

**Remarks.**Thach (2020) stated that “…sinistral shell, elongate aperture extending leftward and very far from shell axis” are the diagnostic characters. However, these are synapomorphies of the sinistral-*Hemiplecta* clade. Recent systematic revision showed no evidence of the unique phylogenetic subdivision and, therefore, recognized this name as a junior synonym of *H. salangana* (see Sutcharit et al. 2021).

## Results and conclusion

Nine valid species of the genus *Hemiplecta* occur in Thailand, five of these are the dextral shell coiling species, and the other four are sinistral shell coiling species. In order to broaden our comparison among species of *Hemiplecta* s.l., we gathered and compared anatomical data from the literature for nineteen species (Table 2). This comparison indicated that fourteen species are likely to have been correctly placed in the genus *Hemiplecta*, but not the other five species. The genital characters are relatively similar among species within this genus, except for the penial verge, penial sculpture, and terminal part of male genitalia (epiphallus, epiphallic caecum and flagellum), which are taxonomically informative at the species level (Table 2). Interestingly, these fourteen species all have a similar gametolytic organ structure: globular gametolytic sac with an undifferentiated duct. A recent systematic study has shown congruence between the traditional morphology-based species taxonomy and the molecular phylogeny (Sutcharit et al. 2021). This indicates that accurate generic recognition can be based on the genitalia character especially the globular shape of the gametolytic sac. However, the critical role of the gametolytic organ other than extracellular digestion of excess reproductive products has never been reported in the stylommatophoran (Goméz 2001; Baur 2010).

This comparison further indicated that the other five species are likely to have been inappropriately placed in the *Hemiplecta* (Table 2). Four species: *H. densa* (Adams & Reeve, 1850) from the Philippines, *H. werberi* (Sarasin & Sarasin, 1899) from Sulawesi, *H. foersteri* Kobelt, 1914 from Papua New Guinea, and *H. belerang* Cilia & Abbas, 2012 from Sumatra exhibit a long gametolytic duct, with or without a dart apparatus (Table 2) and are clearly distinct from the typical characteristics of the *Hemiplecta* (Wiegmann 1898; Niethammer 1937; Wiktor 2003; Cilia and Abbas 2012). On the other hand, these four species have a dextral shell, and the presence of shell lobes suggest a close relationship to the genus *Nanina*. In addition, *H. malaouyi* (Morgan, 1885) from Peninsular Malaysia exhibits a coiled epiphallic caecum, long gametolytic organ, and presence of shell lobes (Table 2), which are the unique characteristics of the Macrochlamydinae (Blanford and Godwin-Austen 1908; Solem 1966; Schileyko 2003). However, the relatively large shell size (width about 40 to 60 mm) is distinct from other known genera within the Macrochlamydinae. Further anatomical information and molecular analyses will elucidate whether the generic placement is appropriate or whether these species form a distinct group.

In the species list, 39 available species-level names are recognized as part of the genus *Hemiplecta* and described from Indochina, including Peninsular Malaysia and Myanmar. There are six nominal species for which the name-bearing type could not be discovered, except the four nominal species: *H. auriettae*, *H. gordonae*, *H. textrina* and *H. theodori* where the topotypic specimen are figured as representative. However, generic placement of many species are still provisional because these species are known only from their shell descriptions without the genitalia characters. Like the other land snail group in Indochina, a systematic revision has never been studied, and species recognition is difficult. The species have long been described with only a brief description and without illustrations of unique characters of the species. This species list with illustrated type or authentic specimens provides a key species data and facilitates proper species identification.

## Supplementary Material

XML Treatment for Hemiplecta

XML Treatment for Hemiplecta humphreysiana

XML Treatment for Hemiplecta distincta

XML Treatment for Hemiplecta funerea

XML Treatment for Hemiplecta esculenta

XML Treatment for Hemiplecta nemorosa
